# Hydrogen and biomass-based carbon source integration for iron and steel manufacturing: A systematic review of Life Cycle Assessment studies

**DOI:** 10.12688/openreseurope.20725.2

**Published:** 2026-02-03

**Authors:** Nethmi Sewwandi Kankanamge Dona, Ana Arias, Franco Donati, Stefano Cucurachi, Rene Kleijn

**Affiliations:** 1 Institute of Environmental Sciences, Universiteit Leiden Centrum voor Milieukunde, Leiden, South Holland, 2300 RA, The Netherlands

**Keywords:** Life Cycle Assessment, Iron, Steel, Hydrogen, Biomass

## Abstract

Iron and steel manufacturing is a material-intensive, energy-intensive, and emission-intensive process that is focused on attaining carbon neutrality. An important step towards decarbonizing iron and steel manufacturing is quantifying the environmental impacts associated with its potentially sustainable emerging technologies. In this study, we conducted a systematic review of 21 Life Cycle Assessment (LCA) studies that integrated hydrogen and/or biomass in iron and steel production. We categorized various technologies following an LCA approach, focusing on the technological and regional definition of goal and scope and impact categories of Global Warming Impact (GWI), Terrestrial Acidification (TA), Fossil Resource Scarcity (FRS), Mineral Resource Scarcity (MRS), and Fine Particulate Matter Formation (FPMF). According to the findings, GWI of steel ranges from -845 kg CO _2_ eq. to 2287 kg CO _2_ eq. per ton of steel and the GWI of iron ranges from -41kg CO _2_ eq. to 2799 kg CO _2_ eq. per ton of iron. Furthermore, the integrated technologies also have corresponding average approximate TA, FPMF, MRS, and FRS of 11 kg SO _2_ eq., 3 kg PM 2.5 eq., 83 kg CU eq., and 304 kg oil eq. per ton of iron. The variations in these results are highly dependent on technological and regional differences of the goal and scope of the studies. This study reinforces the significance of exploring hydrogen and/or biomass integration methodologically, linking results to various LCA choices. Additionally, the results derived from this review also aim to emphasize the need for technological and region specific modelling, data and methodological standards, transparent reporting in conducting LCA in integrating hydrogen and/or biomass into the iron and steel industry.

## Plain Language Summary

This systematic review aims to discuss the application of Life Cycle Assessment (LCA) to iron and/or steel manufacturing technologies where hydrogen and/or biomass is integrated into their manufacturing processes. The decarbonization of the steel industry is considered to be of paramount importance as it emits 2.21 billion tons of CO2 annually. Therefore, the use of hydrogen and/or biomass is a viable alternative towards a low-carbon iron and steel industry. LCA is one among many sustainability assessment techniques that can be used to assess the environmental profiles of emerging technologies, and in our case, the hydrogen and/or biomass integration.

According to the findings of our review, hydrogen can be integrated as a reducing agent, and biomass can be integrated as an energy source or as an alternative carbon source that can replace fossil coal fully or partially in iron and steel manufacturing. Hydrogen itself can be integrated in several proportions, such as 70%, 75%, or 100%, and it is expected to lower the CO2 emissions approximately by 90%, which is 150kg of CO2 per ton of steel. On the other hand, the integration of biomass-based sources is expected to generate negative emissions as the biomass sources are considered biogenic in most cases.

Our review is expected to provide an aggregate and a fair comparison of the emissions and environmental impacts associated with hydrogen and/or biomass integration in iron and/or steel using several impact indicators. It is also aimed at encouraging the use of LCA guidelines in a non-biased, optimal way to compute the emissions of the iron and steel industry. It is also expected to support policy decision-making and guide LCA practitioners towards relevant resources in conducting LCA studies associated with integrated and emerging technologies in the iron and steel sector.

## Introduction

The manufacturing of iron and steel accounts for 9–11% of the global greenhouse gas (GHG) emissions ( Zhang *et al*., 2023). In 2020, every ton of steel that was manufactured emitted an approximate average of 1.89 tons of CO _2_ into the atmosphere ( Mandova, 2019). In order to contribute towards the below 2 °C limit, the iron and steel industry is expected to retain its CO _2_ budget at 50 gigatons from now till the year 2050 ( IPCC, 2022; Tian *et al.*, 2018). On the other hand, along with the economic progress, the cumulative demand for iron and steel is expected to rise as the Iron and Steel Industry (ISI) is expected to represent the backbone of the green economy with renewable infrastructure and clean transportation ( Petrescu *et al*., 2019; Xi *et al.*, 2021). Thus, as a sector with identified hard-to-abate emissions and a rising demand, several novel steel and iron-making technologies are being researched extensively to accelerate the decarbonization of the sector ( Bataille *et al*., 2018). These technological innovations must be readily coupled with relevant sustainability assessment techniques and evaluated based on their technological, regional differences in goal and scope definition and environmental indicators to understand the contribution of such to the decarbonization endeavors of the ISI ( Durga *et al.*, 2024; Schinkel *et al.*, 2022).

The iron and steel production is an intricate process that varies regionally due to differences in raw material availability, energy supply, and technology, and several other factors ( Sekiguchi, 2019; Xylia *et al.*, 2018). This absence of a one-size-fits-all decarbonization solution has resulted in different regions launching tailored low-carbon emission projects that suit their Best Available Technologies (BATs) ( Chan *et al*., 2019). For example, the Ultra Low CO _2_ Steelmaking (ULCOS) project assessed the four most promising technologies for the European region, which analyzed the use of recycled top gas, coke-free iron and steel, fast melting direct reduction, and electrolysis in the ISI ( Bataille *et al*., 2018). Similarly, the Japanese project Cool Earth 50 (COURSE50) is targeted at reducing emissions by using hydrogen as a reducing agent ( Zhao & Dong, 2018). Several other projects, such as POSCO in South Korea, COREX and HIsmelt in China, are also focused on materializing the iron and steel decarbonization targets in their respective countries ( Quader *et al.*, 2015; Zhang *et al.*, 2021a). Therefore, in-depth studies must be conducted to assess and compare the impact of technological and regional heterogeneity on the sustainability of the ISI, especially in the use of hydrogen and biomass, which are relatively novel integrations Xu & Lin, 2016). Thus, as the first research objective of this study, we intend to identify the technological and regional differences in the goal and scope definition of hydrogen and/or biomass-based steel studies.

In particular, experts regard hydrogen as a promising carbon-substituting, alternative reducing agent that can partially or fully replace conventional coal and fossil fuels ( Egerer *et al.*, 2024; Pitsch, 2024). Hydrogen can be found in abundance in fossil-based sources such as natural gas, and it can also be manufactured from renewable sources such as wind, solar, geothermal, or biomass energy ( Gielen *et al.*, 2019; Islam *et al*., 2024). Hydrogen also has a high calorific value and a higher reaction rate, along with thermal conductivity, which enhances its potential as a reducing agent in iron and steel manufacturing ( Liu *et al*., 2021). In previous research, we can see the utilization of hydrogen in ISI in Blast Furnace (BF), Direct Reduced Iron (DRI), smelting reduction, and other ancillary procedures ( Ranzani Da Costa *et al.*, 2013).

The decarbonization potential of hydrogen-based ISI also depends on the hydrogen-producing technology and the source of electricity used to produce hydrogen ( Neuwirth *et al.*, 2022). Hydrogen-producing technologies such as Coke Oven Gas (COG) reforming, Steam Methane Reforming (SMR), and coal gasification, which are conventionally termed as emission-intensive, have shown promising improvements and will likely provide crucial support in decarbonization with renewable electricity plug-ins ( Li & Cheng, 2020; Muritala *et al.*, 2020). Additionally, recent developments in technologies such as hydrogen production using water electrolysis, integrated with renewable energy, hydrogen production through biomass gasification, and hydrogen production using nuclear technology may further contribute to decarbonization efforts through their integration into iron and steel manufacturing ( Boretti, 2020; Doranehgard *et al.*, 2017). Thus, it can be identified that utilizing green hydrogen produced using renewable sources can contribute significantly towards decarbonizing the ISI by fully decarbonizing the source of electricity used for hydrogen generation ( Watari & McLellan, 2024).

Similarly, biomass can also be recognized as a promising decarbonizing agent due to its abundance, variety, and biogenic nature in emissions ( Wang & Wu, 2023b). Developments in biochemical refineries, which include fossil fuel substitutes such as hydrogen and bio-methane, can be identified as significant contributors to the establishment of the bioeconomy in Europe ( Song *et al*., 2021). Though several pretreatment techniques, such as thermal upgrading, are required for the further processing of biomass, they can still be enhanced to produce materials that can be used in metallurgical applications ( Khasraw *et al.*, 2024; Wei *et al.*, 2024). Biomass-based materials can be used as reducing agents, charging materials, or injections into the ISI ( Nwachukwu *et al.*, 2021). Specifically, the partial or full replacement of fossil-based inputs with renewable biomass-based products appears to exhibit economic and technical feasibility in the decarbonization of the iron and steel sector ( Nwachukwu *et al.*, 2022).

The possibilities of alternative biomass-based carbon sources, such as rubber and palm shells, and forest waste, charcoal, or coffee residues, being integrated into ISI have been researched intensively ( Farhana Diyana Mohd Yunos *et al.*, 2015; Jahanshahi *et al*., 2015; Mousa *et al.*, 2016). Previous studies have also indicated that biomass integration in European iron and steelmaking should be seriously considered as long as it can be sustainably sourced in between regions ( Swennenhuis *et al.*, 2022).

All these technologies and solutions must be assessed to quantify their environmental impacts. Life Cycle Assessment (LCA) is the methodology that is used frequently for this purpose ( Finnveden *et al*., 2009). LCA is a framework that allows assessing the environmental impacts linked with product systems or processes, and it is standardized by the International Organization of Standardization (ISO) in ISO 14040 and ISO 14044 ( International Standard Organization, 1997). LCA ensures the quantification of the impacts on the environment from a life cycle point of view, from cradle to grave ( European Commission, 2010). Thus, as a second research objective of our study, we have compiled and compared multiple hydrogen and/or biomass steel scenarios and LCA indicators to understand the environmental impacts of these novel integrations.

Several articles and review articles on hydrogen and/or biomass use in ISI can be found in the literature ( Bilby & Hewitt, 1962; Burchart-Korol, 2013; Su *et al*., 2022). These articles are fundamentally focused on exploring the metallurgical subject matter associated with integration. For example, several studies have carried out simulation-based assessments of integrating hydrogen and/or biomass into steel, focusing on material and energy efficiencies under various technological configurations ( Atrens *et al*., 2020; Bilby & Hewitt, 1962; Oriani, 1970). We also found multiple studies that assessed the general sustainability of the hydrogen and/or biomass-based ISI. These studies focused on concepts such as the carbon footprint of hydrogen-based primary steel production, the policy implications of the hydrogen and biomass economy in the steel sector, the use of eco-efficiency to compute the impact of biomass use in steel making, and applying LCA to a single or multiple hydrogen and/or biomass-based steel configurations ( Bhaskar *et al.,* 2022; Sambandam *et al.,* 2022; Suer *et al.,* 2022a; Suopajärvi *et al*., 2018).

It is important to note that we found a notable absence of a comprehensive systematic review that covers LCA studies of integrated hydrogen and/or biomass technologies in the literature. Therefore, our work's novelty lies in its provision of a platform for comparing integrated technologies based on their key technological and geographical features and using several impact assessment indicators.

Therefore, we pursue the objectives of this study using 3 steps : 1) analyzing the use of LCA in the assessment of hydrogen and/or biomass-integrated iron and steel manufacturing in a technological matrix 2) analyzing the use of LCA in the assessment of hydrogen and/or biomass-integrated iron and steel manufacturing in a regional matrix 3) to compare available scenarios from hydrogen and/or biomass steel based LCA case studies using an impact assessment matrix 

## Methods

This study analyzes the current research on the environmental impacts of using hydrogen and/or biomass in the ISI. Therefore, we review the relevant literature focusing on the following main research question: ***What is the current state of the art in applying LCA to assess the environmental impacts of hydrogen as a reducing agent and/or biomass as an alternative carbon source in iron and steel manufacturing?***

This main research question is addressed through the sub-research questions:

**
*1. *
**  ***How do studies compare from a technological perspective in their goal and scope definition?***
**
*2. *
**  ***How do studies compare from a regional perspective in their goal and scope definition?***
**
*3. *
**  ***How do the scenarios in the studies compare in their impact assessment results? ***

To answer these research questions, we use *the Preferred Reporting Items for Systematic Reviews and Meta-analyses (PRISMA)* guidelines ( Moher *et al.*, 2009). The PRISMA guidelines allow us to specify several criteria of the literature that fall within the scope of the review. Following the guidelines on the PRISMA protocol approach, this review assesses the LCA applications in hydrogen and/or biomass integration in iron and steel case studies. Thus, this review extracts information from the LCA studies following the ISO standard on LCA, i.e. the goal and scope definition, and life cycle impact assessment results, focusing especially on the downstream and upstream processes of ISI ( Finkbeiner *et al.*, 2006).

### Procedure of the review

First, we conducted the search using the Web of Science (WoS) and Scopus ( Mongeon & Paul-Hus, 2016). Both databases are well-known scientific citation indexes and provide access to multiple LCA-based journals ( Gaurav *et al*., 2021; Hou *et al.*, 2015). Both databases also provide a function where the exact search queries and filtering can be applied, enhancing the transparency and the reproducibility of the work ( MacFarlane *et al.,* 2022; Pranckutė, 2021). Google Scholar was avoided due to its over-inclusivity and major overlaps with the WoS and Scopus ( Meho & Yang, 2006). 

We collected articles from 2014 to 2024 using the following keywords: ***TS= ("LCA" OR "Life Cycle Assessment") AND ("Steel" OR "Iron") AND ("Hydrogen" OR "Biomass" OR "Biochar" OR "Charcoal")*** where TS refers to ‘Topic’ that is searched in the title, abstract, authors, and keywords. This search string was compiled based on the synonyms identified from previous work. We tested the comprehensiveness of the search string by checking if key known papers will appear once searched. We were focused on extracting information only from work that followed the ISO 14040 and ISO 14044 framework ( Guinee, 2002). Therefore, alternative sustainability-related terminology, such as carbon footprint, carbon accounting, was excluded from the analysis.

The search included only the peer-reviewed articles and excluded review articles, conference articles, and proceedings, and articles that were not written in English. Grey literature was excluded to maintain the standard of including only scientifically peer-reviewed work. This included policy papers and reports. These exclusions increased the relevance, consistency, and comparability of our study further.

**Figure 1.**
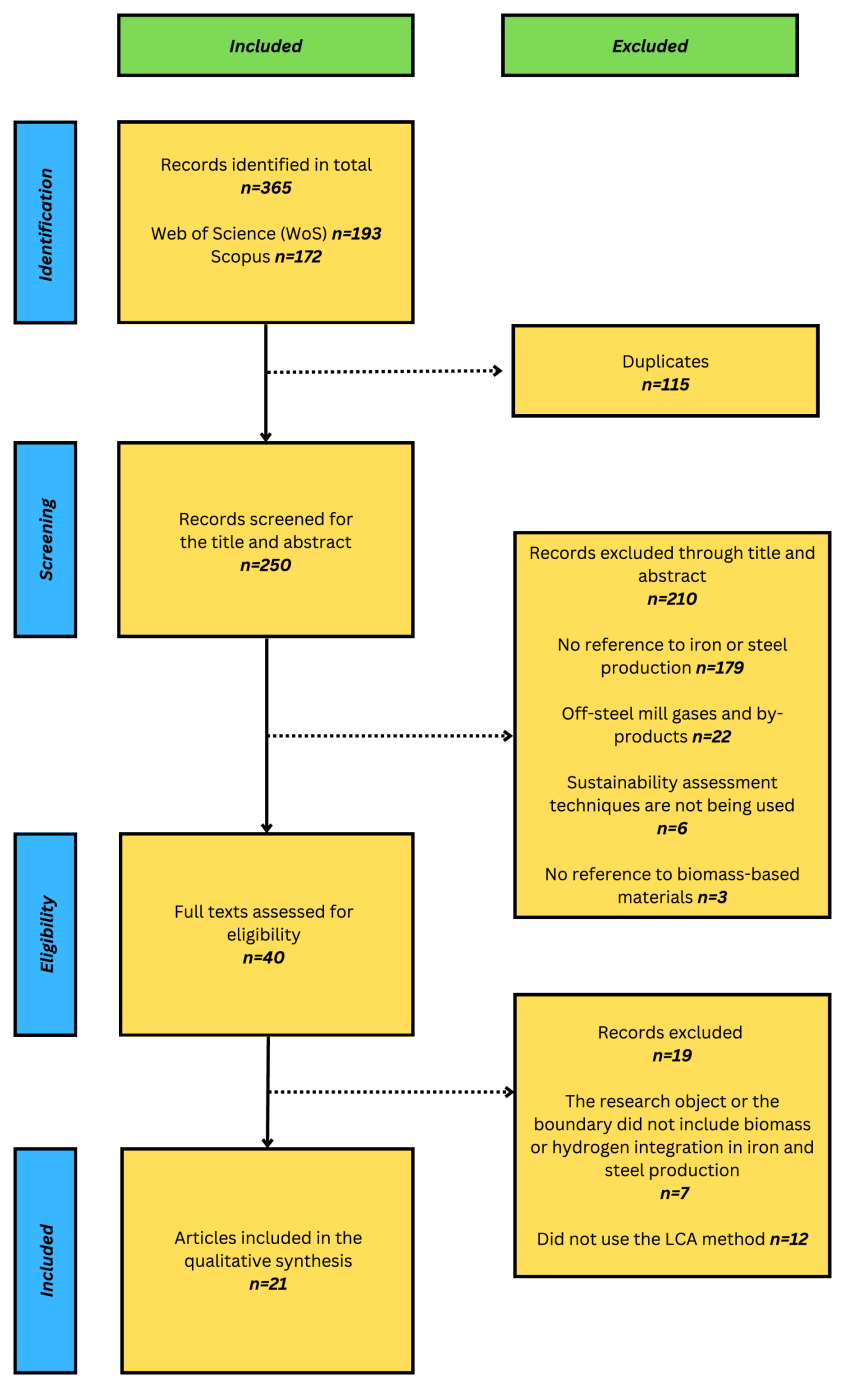
The screening process based on the PRISMA framework. The PRISMA flowchart (LCA: Life Cycle Assessment).

This initial search generated a total of 365 articles (193 WoS; 172 Scopus). After removing duplicates (115), we had 250 records, which were then taken further into the screening process. A flowchart of the process of screening based on PRISMA is shown in Figure 1.

The screening process included title and abstract screening. This title and abstract screening resulted in the exclusion of 210 articles. The exclusion criteria were: did not refer to iron and steel production ***(n=179)***, discussed the off-steel mill gases and by-products ***(n=22)***, did not concern sustainability assessment studies ***(n=6)***, or did not refer to biomass ***(n=3)***.

Then, 40 articles were analyzed for eligibility using the following inclusion criteria, of which 19 articles were removed: 1) The research object or the boundary did not involve biomass or hydrogen ***(n=7)***, and 2) did not use LCA ***(n=12)***. These 12 papers used techniques such as Techno-Economic Assessment (TEA), Material Flow Analysis (MFA), Hybrid Life Cycle Assessment (HLCA), Input-Output Analysis (IOA), and Social Life Cycle Assessment (SLCA). The HLCA publications were excluded as it is a combination of IOA and process-based LCA, and the SLCA studies were excluded as the impact categories were centered on social impact indicators that fall outside the scope of this review.

All records were screened by a single reviewer (the first author) following these pre-defined exclusion and inclusion criteria. The screening process of the articles can be accessed at the extended data for further clarity. 

Finally, 21 literature articles were obtained for the qualitative and quantitative synthesis. The search was completed on the 4 ^th^ of December 2024. A quality appraisal of the chosen studies was conducted using the Life Cycle Initiative standards, and the full matrix of this can also be found in the extended data ( Baitz, 2019).

## Results

In this section, we first discuss the technological scope of the case studies using a glossary of terms to interpret the results presented in the literature. This will assist the reader in understanding the technical terms discussed within the text. Then, we present an analysis of the differences in the choices of defining goal and scope in the literature depending on the region. This will address the first two research questions of our study, where the reader will understand how studies compare from a technological and regional perspective in their goal and scope definition. Afterward, we compare the impact assessment results that were found in the analyzed literature in order to address the third research question of our study, where the reader will understand how the impact assessment results from the explored scenarios in the studies compare

A separate methodological matrix of how data was gathered, under what criteria for this results section can be accessed at the extended data for further reference.

### The technological differences in the goal and scope definition

The assessed case studies refer to novel and emerging technologies where hydrogen and/or biomass integration in ISI is discussed. The readers must be able to identify and differentiate between the varying forms of hydrogen and biomass that will be integrated as inputs into the ISI. Depending on the form of hydrogen and biomass, the impact assessment results can be vastly different from each other. Similarly, the iron and steel output can be different depending on its form and final composition, affecting the impact assessment results in varying degrees. Table 1 will provide an overview of the concepts that we will discuss in this article, along with their definitions, improving the readability of the article.

**Table 1.** Glossary of terms. Glossary of terms to enhance the readability of the work.

Term	Definition
**Charcoal**	It is carbonized wood that is primarily used as a reductant or fuel. Can exist in the form of lumps or fines ( Lehmann & Joseph, 2015).
**Biochar**	It is manufactured from biomass that is sustainably sourced and can be used in industrial processes such as carbon sequestration or as a fuel ( Hagemann *et al*., 2018).
**Pellets**	They are a processed form of iron ore, specifically designed for direct use in Blast Furnace (BF) or in direct reduction plants ( Katrak, 2001).
**Hydro char**	It is a material produced through Hydrothermal Carbonization (HTC) at temperatures between 180–250 °C ( Rodríguez-Narvaez *et al.*, 2022).
**Torrefied biomass fines**	Torrefaction is a thermal treatment technique for biomass to thermally degrade organic materials in a temperature range of 200–300 °C ( Zakaria *et al.*, 2023).
**Direct Reduced Iron (DRI)/ ** **Sponge Iron (SI)**	The output of the direct reduction process ( Nassaralla, 2001).
**Hot Briquette Iron (HBI)**	It is a form of DRI that gets compacted at temperatures higher than 650° C and with a density that is higher than 5,000 kg/m ^3^ ( Bersenev *et al*., 2022).
**Hot Metal (HM)**	The molten iron produced in the BF ( Zhou *et al*., 2020).
**Hot-rolled steel/ Hot ** **Rolled Coil (HRC)**	Steel that is preheated to a high temperature (which is generally at 850–1200 °C) and is continuously rolled between two rotating cylinders ( Samarasekera, 2001).
**Crude steel**	The solid state of steel after melting, which can either be processed or sold ( Bailera *et al.*, 2021).
**Pig iron**	The output from iron ore smelting with a carbon-intensive fuel such as coke ( Bailera *et al*., 2021).
**Liquid Steel (LS)**	It is the immediate hot molten steel output from steel melting. It can be further cast into ingots ( Richardson *et al.*, 1994).
**System boundary**	It refers to the boundaries within which the Life Cycle Assessment (LCA) is being carried out ( Khatri & Pandit, 2022).
**Cradle to gate**	It refers to all processes from the material extraction phase to the factory gate ( Cao, 2017).
**Functional Unit (FU)**	It is a function that is being defined through technical requirements associated with the product system to be quantified as a quantity, quality, or duration ( Tait & Cheung, 2016). FU can be used as the basis for comparing products ( Furberg *et al.*, 2022).
**Global Warming Impact** ** (GWI)**	This measures the potential global warming from airborne greenhouse gases (GHG), and it is measured using the unit kg CO _2_ eq ( Edenhofer *et al*., 2015).
**Fine Particulate Matter** ** Formation (FPMF)**	This refers to the release of fine particulates at ground level, adversely affecting human health ( Sangkham *et al*., 2024). It assesses the damage to human health caused by Particulate Matter (PM). PM exists in the form of PM2.5 and PM10. It is measured using the unit PM 2.5 eq. in this study.
**Terrestrial Acidification** ** (TA)**	It measures the chemical property changes in soil with nutrient deposits such as nitrogen and sulfur in the form of acids ( Azevedo *et al.*, 2012). It is measured using the unit kg SO _2_ eq. in this study.
**Mineral Resource Scarcity ** **(MRS)**	This measure computes the inability of the resources that are of a mineral base to meet the demands of its environment ( Drielsma *et al*., 2016). It is measured using the unit kg CU eq.
**Fossil Resource Scarcity ** **(FRS)**	It computes the extent of the availability of fossil fuel-based resources ( Arvidsson *et al.*, 2021). It is measured using the unit kg oil eq.

### Regional differences in goal and scope definition

**Figure 2.**
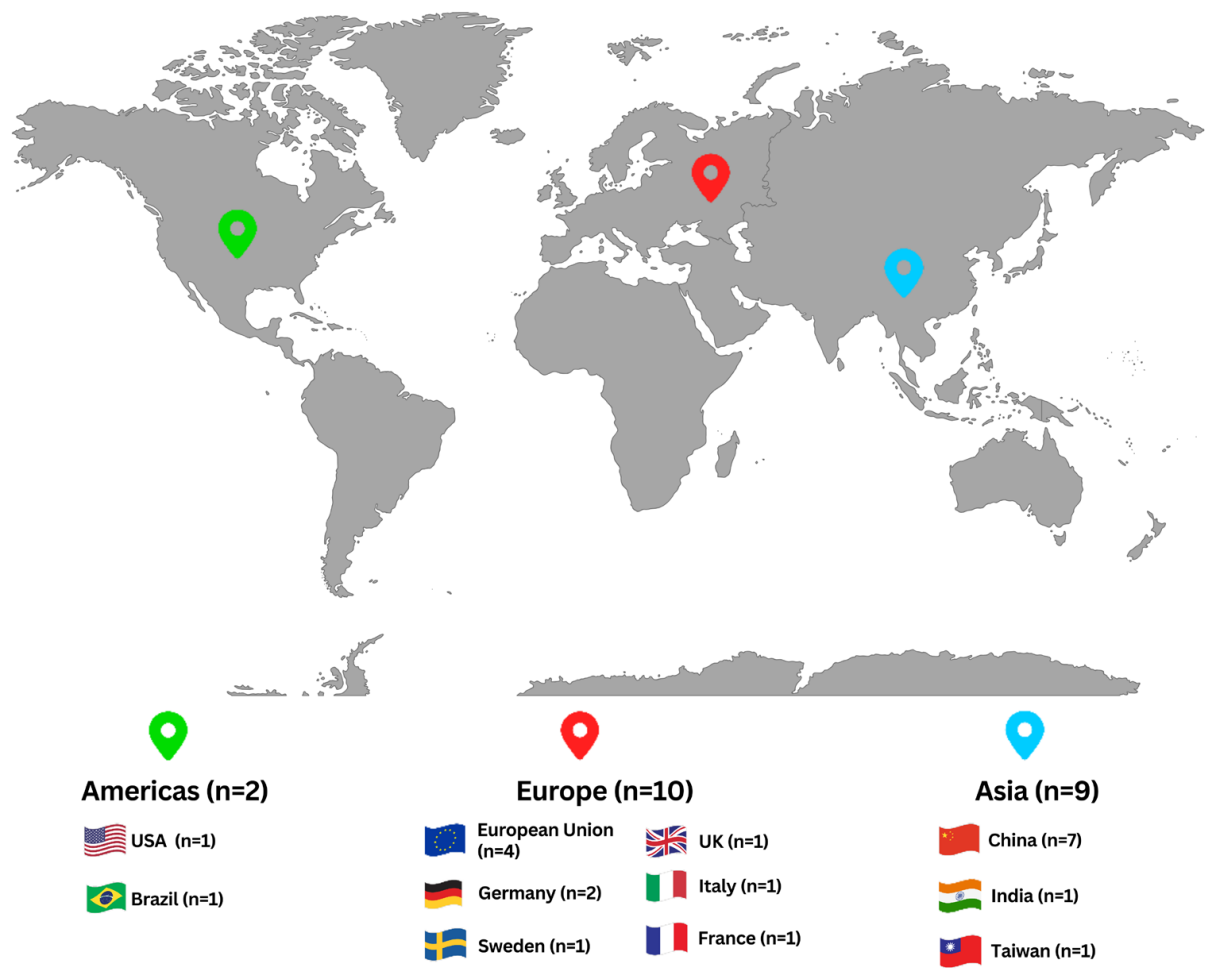
The spatial distribution of the case studies. The geographical area of the case studies found in the literature.

***Asia.*** As shown in Figure 2, the Asian case study distribution includes studies from China (7 case studies), Taiwan (1 case study), and India (1 case study). All Asian case studies have conducted cradle-to-gate assessments for ISI. The studies that focused on steel production have chosen the functional units of one ton of hot-rolled steel and one ton of crude steel for their LCAs.

Among the Chinese case studies, we observed the use of Hydrogen-enriched shaft direct reduction methodology (HSE process) in integrating hydrogen to produce Electric Arc Furnace (EAF) steel. The study was conducted for the data collected between 2015–2022, and it is expected to have an energy consumption of 9.08 GJ/t, resulting in a 53.75% energy saving compared to the BF-based steel. It is also expected to generate CO _2_ emissions worth 1079.56 kg CO _2_ eq./t, creating a 47.45% emission reduction in comparison to the BF based steel making ( Li *et al*., 2022a). Additionally, the importance of the renewability of the hydrogen manufacturing source is being highlighted through the HSE process’s integration with renewable electricity-based water electrolysis hydrogen to generate low carbon emissions. The electrolysis-based hydrogen steel has 120.92 kg of CO _2_ emissions less compared to the coal gasification-based hydrogen EAF steel (HESE process) ( Li *et al*., 2022b). Also, the use of Supercritical Water Gasification (SCWG) with HSE, is expected to reduce emissions by 28 % compared to the steel manufactured through coal gasification-based hydrogen. SCWG is a technique that involves gasifying wet biomass to generate hydrogen as a combustible gas ( Shahabuddin *et al.*, 2020). Similarly, it is expected to be 30 % less emission-intensive compared to the natural gas (NG) based hydrogen EAF steel process ( Huang *et al.*, 2024). Along with the source of hydrogen production, the combined hydrogen supply chain modeling (HSC) indicates that the hydrogen-based route can result in a GHG decline of 10% compared to the fossil-based steel manufacturing route ( Ren *et al.*, 2023).

The case studies that quantify the environmental impacts associated with iron production use functional units such as one ton of hot metal (HM) and one ton of sponge iron (SI). The identified case study in the literature discusses the use of carbonaceous pellets produced from waste wood in hot metal production. In this particular case study, the use of biomass-based pellets to reduce the use of fossil resources in the manufacturing of sinter and iron ore generates emission reductions worth 278 kg CO _2_ eq./t of iron. This emission reduction is for a 30% replacement ratio of sintered ore using biomass-based pellets ( Lu *et al*., 2024).

The emission mitigation potential in the use of Palm Kernel Shell Charcoal (PKSC) in the SI production process using the rotary kiln is being discussed in the Indian case study. The use of PKSC assists in attaining negative GHG emissions worth −41 kg CO _2_ eq/t of iron. However, the impact on land and water usage, and food security should be assessed in a case where PKSC will be harvested from palm tree cultivations, to minimize the burden-shifting ( Vigneswaran *et al*., 2024). The case study that explores the use of Mango Pit Biochar (MPB) showed a 220 kg CO _2_ eq./t of HM emission reduction in a scenario where MPB replaces Pulverized Coal (PC) by 100% ( Yang *et al*., 2024).

Substituting charcoal for coke and anthracite in the BF, as well as the use of wood pellets as a fuel in reheating furnaces, have also been studied as potential biomass-based decarbonization options for ISI. The charcoal replacement of 20% coke and 100% anthracite in the BF appears to minimize the CO _2_ emissions by 14% for a ton of hot rolled steel, according to the results of a Chinese case study. These results are based on the data collected for the inventory in 2017 ( Liang *et al*., 2020). Similarly, using wood pellets as a heating fuel in the EAF indicated that wood pellet fuel only results in 19.3 kg CO _2_ eq. per ton of iron, whereas the heavy fuel oil would emit 129.6 kg CO _2_ eq. per ton of iron ( Lin *et al*., 2016).

***Europe.*** In the literature presented, 10 case studies discussed the ISI in Europe. Europe is the second biggest iron and steel manufacturer in the world, accounting for 11% of the global iron and steel output ( Vögele *et al.*, 2020a). ISI in Europe is also a major subsector in its employment and value addition to economies, making it an important geographic area of investigation ( Vögele *et al.*, 2020b).

All European case studies have conducted cradle-to-gate LCAs. The case studies that quantified the environmental impacts have mostly utilized the functional units of one ton of steel, Hot Rolled Coil (HRC), Liquid Steel (LS), and molten steel except for one kilogram of steel in rare cases.

The integration of hydrogen in steel production has been discussed in its use in the DRI-EAF and BF. Additionally, the use of hydrogen-based Hot Briquetted Iron (HBI) in BF, hydrogen as a reducing agent, and hydrogen as a fuel-switching option that replaces coal and NG are some of the decarbonization options that have been discussed in the literature.

As the sustainable European grid mix forecast for 2040 indicates, the steel manufactured via the hydrogen-DRI-EAF route in 2040 will have a GWI of 0.75 kg CO _2_ eq./kg steel ( Suer *et al.*, 2022a). According to the literature, the use of green hydrogen and renewable energy will be the sole factor that would lead to manufacturing low GHG footprint steel, resulting in less than 600 kg CO _2_ eq./t of crude steel ( Souza *et al.*, 2023). Also, the utilization of hydrogen in a BF reduces 9% of the emissions, and a 10%-17 % reduction potential can be seen through the use of hydrogen-based HBI within a BF ( Suer *et al.*, 2021). According to the projected LCA results from a German case study, hydrogen is expected to contribute to a decline of direct emissions by approximately 96 % in comparison to traditional steel manufacturing in 2050 ( Weckenborg *et al.*, 2024). However, a UK case study presents that hydrogen will have adverse impacts on ecotoxicity and metal depletion despite its contribution to GHG reductions ( Cooper & Hawkes, 2024).

Several studies in Europe discussed biomass integration in the form of hydro char and bio-syngas. The use of hydro char manufactured using pruned vine and exhausted grape marc (EGM) can be a potential alternative carbon source in the EAF and may reduce the GWI by 2% from the conventional steelmaking process ( Cardarelli & Barbanera, 2023). The renewable syngas manufactured from biomass gasification, or bio syngas, can have a significant influence on the future energy system sustainability as they can replace fossil gases and require no modification to the existing DRI furnaces to apply it as a reducing agent ( Bolívar Caballero *et al.*, 2022). The GWI for the bio syngas based DRI-EAF is 75% lower than the state-of-the-art NG-based DRI-EAF route. It is also 85% lower than the GWI of the BF-Basic Oxygen Furnace (BOF) route. The bio-syngas-based DRI-EAF is expected to have 251 kg CO _2_ eq./t crude steel from cradle to gate, which is less than the renewable hydrogen-based DRI-EAF route ( Nurdiawati *et al*., 2023).

The environmental impacts of iron production have been quantified with varying functional units such as liquid iron, pig iron, and HM. In the literature, we found studies addressing biomass in iron manufacturing in forms such as lumps, charcoal fines, torrefied biomass fines, charcoal at sintering, and biomass hydro char. These studies prove that substituting 20% of coke with biomass could deliver a decline of 15% in the total GHG emissions and save 300 kg CO _2_ eq./t of pig iron ( Fick *et al.*, 2014). Additionally, the use of hydro char in the ironmaking process would result in a total footprint of 2054 kg CO _2_ eq./t of steel. This GWI is 420.61 kg CO _2_ eq. lower than the GWI of the current fossil-based BF ironmaking ( Liang *et al*., 2023).

***Americas.*** As shown in Figure 2, the case studies in the Americas accounted for 2 of the total studies and discussed the steel production in the US and the pig iron manufacturing in Brazil. The US case on the production of steel shows that when there is an 83% or 100% replacement ratio of renewable hydrogen in NG, DRI technologies can decrease CO _2_ emissions by 57% and 67%, respectively, for one ton of steel ( Zang *et al*., 2023).

The quantification of environmental impacts for the pig iron in Brazil showed that in the ex-ante scenario, the carbon and energy footprint reaches 0.69 tCO _2_ eq./t of pig iron and 4.6 GJ/t of pig iron. This was attained from the use of 100 % sustainable charcoal, including the emissions for rail transportation in the LCA work. These values are also two to five times smaller than the conventional BF pig iron production values ( Leão *et al*., 2023).

### Life cycle impact assessment

In this section, we compare the results from the alternative scenarios assessed in the literature. The alternative scenarios that provided information on the impact categories can be found in Table 2. We chose to compare the following impact categories across the collected studies: Global Warming Impact (GWI), Fine Particulate Matter Formation (FPMF), Terrestrial Acidification (TA), Mineral Resource Scarcity (MRS), and Fossil Resource Scarcity (FRS). All scenarios assessed are cradle-to-gate assessments and were compared according to their functional unit.

**Table 2.** Scenarios for the comparison of the impact assessment results. Scenarios presented and analyzed in Figure 3(a)– Figure 3(f).

Publication	Scenarios assessed	Scenario code
( Liang *et al*., 2020)	Charcoal replacing 20% coke and 100% anthracite in the Blast Furnace (BF)	A2
( Lu *et al*., 2024)	Sinter+ carbonaceous pellets in the BF (1275 kg)	B1
	Sinter+ carbonaceous pellets in the BF (1147 kg+130.6 kg)	B2
	Sinter+ carbonaceous pellets in the BF (1147 kg+123.6 kg)	B3
	Sinter+ carbonaceous pellets in the BF (1007 kg+247.2 kg)	B4
	Sinter+ carbonaceous pellets in the BF (892.6 kg+370.8 kg)	B5
( Liang *et al*., 2023)	20% charcoal lumps that will be loaded at the top of the furnace	C2
	20% charcoal fines (Injected through the tuyeres)	C3
	20% torrefied biomass fines	C4
	50% charcoal at sintering	C5
( Liang *et al*., 2023)	The addition of biomass hydro char during the BF injection process	D2
( Vigneswaran *et al*., 2024)	Feed end coal and discharge end Palm Kernel Shell Charcoal (PKSC) in the rotary kiln	E2
	Feed end PKSC and discharge end coal in the rotary kiln	E3
	Feed end and discharge end PKSC in the rotary kiln	E4
	Scenario E2 is integrated with steel production	E6
	Scenario E3 is integrated with steel production	E7
	Scenario E4 is integrated with steel production	E8
**( Cardarelli & Barbanera, 2023)**	Hydro char-based Electric Arc Furnace (EAF) steel	G2
( Suer *et al*., 2022a)	Hydrogen-based Direct Reduced Iron (DRI) EAF (projected scenario)	I1
( Nurdiawati *et al*., 2023)	Bio syngas-DRI-EAF technology	K1
	Bio syngas-DRI-EAF technology + Carbon Capture and Storage (CCS) scenario	K2
	Hydrogen-based DRI-EAF	K6
( Suer *et al*., 2021)	Hydrogen in the BF	L3
	Hydrogen-based Hot Briquetted Iron (HBI) in the BF	L5
( Leão *et al*., 2023)	Coke in the BF	N1
	Partial use of top gas and slag in the BF	N2
	A blend of carbon and iron input in the BF	N3
	Scenario N1 + Scenario N2	N4
	Prospective scenario with an increased use of co-products and a blend of inputs	N5
( Huang *et al*., 2024)	Supercritical Water Gasification (SCWG) combined with Hydrogen-enriched shaft direct reduction (HSE) EAF steel	T1
( Yang *et al*., 2024)	Mango Pit Biochar (MPB) scenario	U2

GWI can be defined as the potential of greenhouse gases to cause atmospheric overheating over time ( Dodd *et al.*, 2017). Figure 3(a) and 3(b) represent the GWI of steel and iron, respectively. According to Figure 3(a), the GWI of steel ranges from -845 kg CO _2_ eq./FU to 2287 kg CO _2_ eq./FU. This range depends on various technological configurations, materials, and energy efficiencies. According to the scenarios analyzed in Figure 3(a), the bio syngas-DRI-EAF route offers the highest emission reductions, approximately a 75% and 85% decrease from the NG-DRI-EAF and BF-BOF routes. As seen in scenario K2, applying CCS to the bio-syngas-DRI-EAF route generates negative life cycle emissions as it uses bio-energy and permanently stores CO _2_. On the other hand, scenario K1represents a bio syngas DRI-EAF without CCS integrations. This route still generates the lowest positive cradle-to-gate emissions compared to the rest. Scenario K6 represents a classic case where hydrogen is being used in the DRI-EAF route, and it shows an 85% decrease from the NG-DRI-EAF route. Similarly, the use of hydro char and anaerobic digestion-based EAF routes (G2) results in a 2.2% reduction in the GWI compared to the fossil fuel-based route. The addition of carbonaceous pellets to sinter (E6-E8) results in 278 kg CO2 eq./t of HM at a 30% substitution rate, indicating an overall decline in emissions when bio-based sources are utilized. Scenario A2 is another biomass-based example where replacing 20% of the coke and 100% of the anthracite in the BF offers 11.8% worth of emission reductions compared to the BF-BOF. The scenario (T1) where SCWG is used in the HSE results in 803 kg CO _2_ eq./t of crude steel. The scenario L3 is representative of another classic case where hydrogen is being used in the BF, attaining 63% decline in emissions compared to the conventional BF-BOF emissions. Scenario L5 further elaborates that hydrogen used in the HBI form can create more emissions reductions worth 14% compared to a hydrogen injection into the BF. The significance of the renewability of the relevant electricity mix from 2030 onwards has been highlighted for the production of hydrogen in scenario I1, as it will result in a 63% decline in emissions compared to the traditional BF-BOF.

**Figure 3a.**
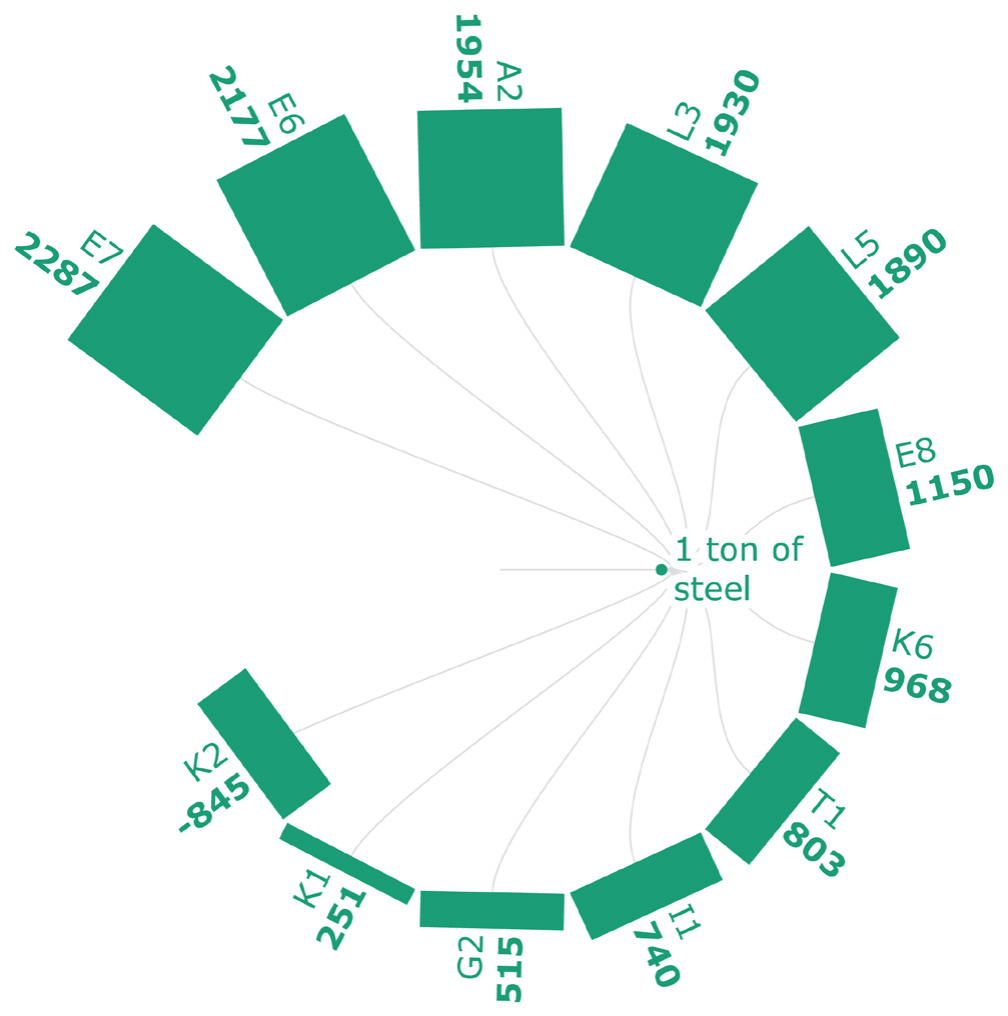
GWI of steel (kg CO _2_ eq./FU). Global Warming Impact of steel (kg CO _2_ eq./Functional Unit). Please refer to Table 2 for the descriptions of the scenarios.

Figure 3(b) represents the GWI of iron production for the selected scenarios. The values in this range from -41kg CO _2_ eq./FU to 2799 kg CO _2_ eq./FU. The scenario N1 is a conventional case where coke is used in the BF, generating 1980 kg CO _2_ eq./FU. This can be used as a baseline to compare the rest of the scenarios with integrations. It also shows that the use of PKSC at both the feed and the discharge end of the SI production process generates a GWI of -41kg CO _2_ eq./t of SI (E4). The negative GWI is a result of the energy that is fully sourced by PKSC, which is carbon-neutral. Scenarios E2 and E3 discuss the use of PKSC either at the discharge end or the feed end. These configurations indicate that, regardless of the technicalities, the use of PKSC generates emission reduction worth by about 200 kg CO _2_ eq./FU compared to the baseline case of using coal instead of PKSC. According to the results of the scenarios from B1-B5, the higher the carbonaceous pellet quantity is, the lower the GWI is. The scenarios from C2-C5 show that the use of torrefied biomass fines reduces the emissions by 14.7% compared to the fossil-based iron. It is closely followed by the pulverized biomass char injection emission reduction, which is 14.5% from the fossil-based iron production. The use of hydro char (D2) reduces the GWI by 420.61 kg CO _2_ eq. compared to traditional BF ironmaking, affirming the positive impact of the use of bio-based sources for emission reductions. It is important to understand that not all biomass-based integrations generate negative emissions. The negative value can only arise from the biogenic nature of the biomass source and crediting full carbon-neutrality in the biomass model. For an example, MPB (U2) replacing PC generates a total of 2220 kg CO _2_ eq./t of HM. Even though it representsa 9–10% emission reduction from the PC-based HM production ( Yang *et al*., 2024), the inclusion of upstream biomass pretreatment processes and the absence of biomass crediting has generated a higher GWI value

**Figure 3b.**
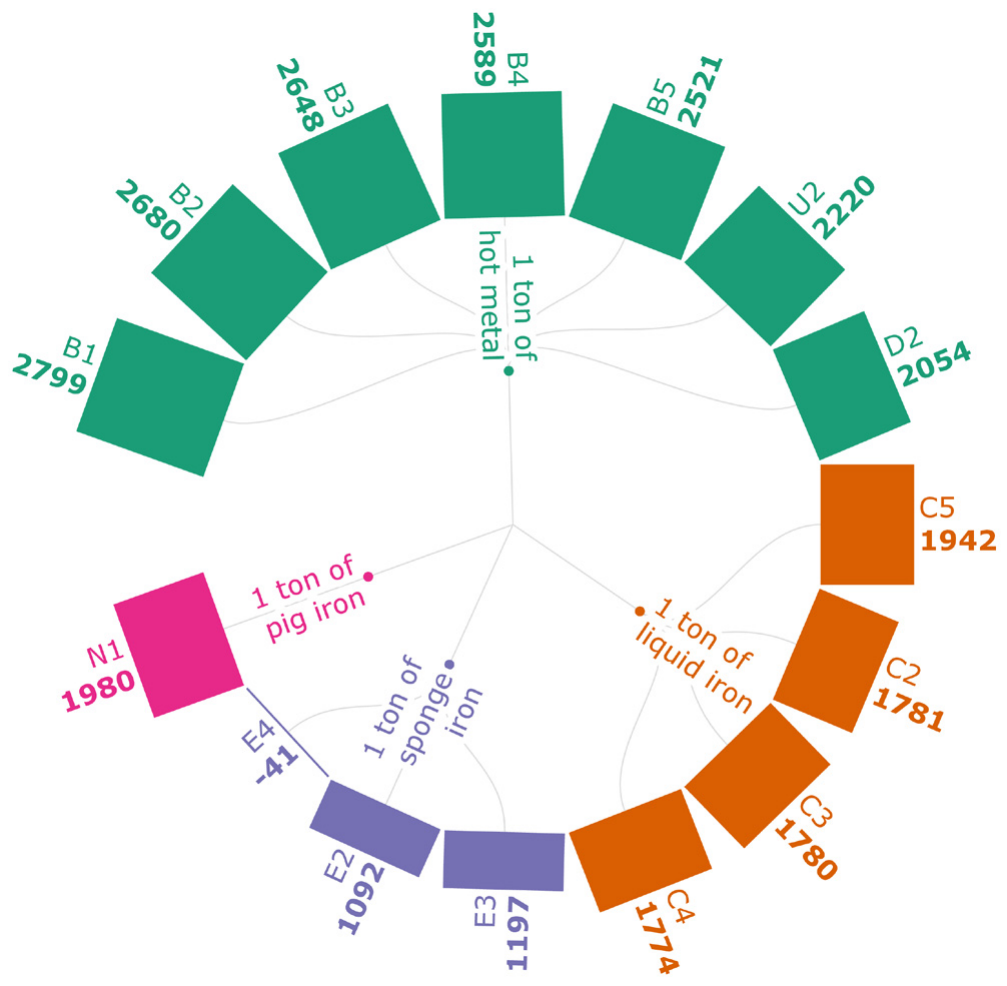
GWI of iron (kg CO _2_ eq./FU). Global Warming Impact of iron (kg CO _2_ eq./Functional Unit). Please refer to Table 2 for the descriptions of the scenarios.

FPMF can be defined as the release of fine particulate matter at the ground level ( Zhang *et al.,* 2022). We analyzed the release of PM 2.5 in our current study. The values in this FPMF analysis range from 0.6 PM 2.5 eq./FU to 5.1 PM 2.5 eq./FU. The analysis in Figure 3(c) shows that the particulate emissions can be reduced by 7.84% when 30% of the sintered ore is replaced with biomass-based pellets. The sintering process, which consumes iron ore, generally contributes the most to the particulate emissions, which is about 69.2 % of the FPMF per ton of HM. Also, the scenarios E2-E4 show that the use of biomass-based pellets can result in reduced particulate emissions despite their technological configurations. 

**Figure 3c.**
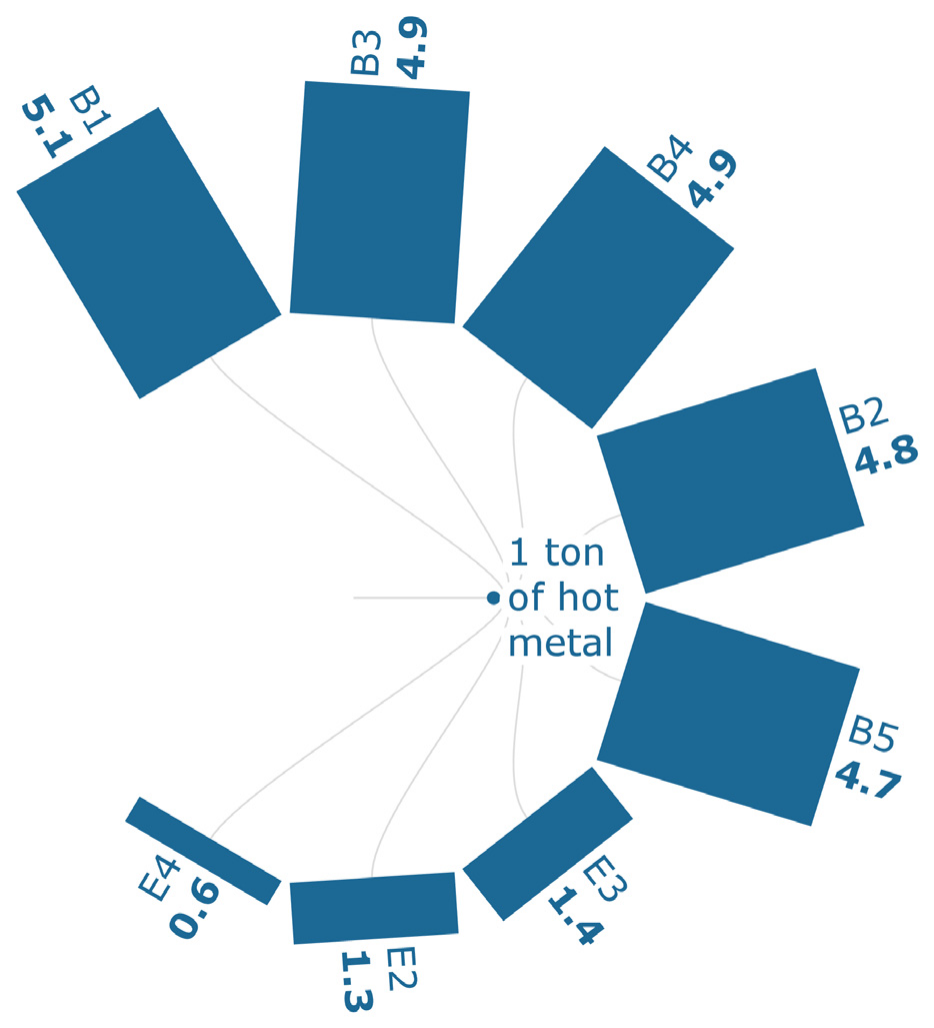
FPMF of iron (PM 2.5 eq./FU). Fine Particulate Matter Formation of iron (PM 2.5 eq./Functional Unit). Please refer to Table 2 for the descriptions of the scenarios.

TA can be defined as the changes in the soil chemical properties depending on the deposition of nutrients such as nitrogen or sulfur in acidic forms ( Zama *et al.,* 2022). The TA values range from 0.2 kg SO _2_ eq./FU to 36.9 SO _2_ eq./FU. The TA levels in scenario D2 of Figure 3(d) indicate that PC injection into the BF has the highest TA at 37 kg SO _2_ eq./t of iron, and it is only 8.6% less than the baseline. The results can be directly attributed to the generation of high-sulfide emissions from the sintering process and the phosphide emissions that emerge from coking and ironmaking processes. Scenarios B1-B5 indicate that the TA is being heavily influenced by the sintering process (77.7 %), followed by the coke-making process (9.5 %). The replacement of sintered ore with biomass-based pellets (E3-E4) indicates that the biomass-based pellets generate a 12.6 % emission reduction per ton of HM with a 30 % substitution rate. Biomass substitution reduces TA to a great degree, as it contains lower sulfur content and can be used to partially or fully replace coke consumption, which can otherwise be a major contributor to the TA levels of iron manufacturing.

**Figure 3d.**
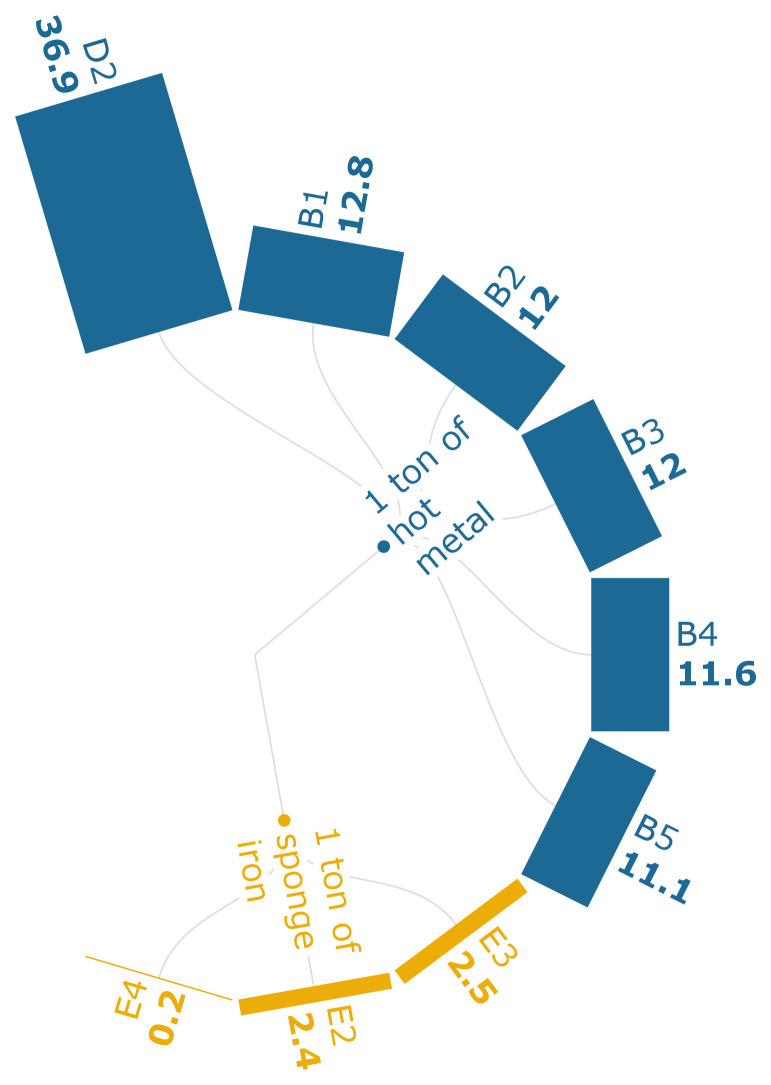
TA of iron (kg SO _2_ eq./FU). Terrestrial Acidification of iron (kg SO _2_ eq./Functional Unit). Please refer to Table 2 for the descriptions of the scenarios.

MRS can be defined as the limited availability of valuable minerals ( Ponomarenko *et al.,* 2021). The MRS values in this analysis range from 79.9 kg CU eq./FU to 87 kg CU eq./FU. In Figure 3(e), B scenarios show that the MRS of the use of biomass pellets is lower compared to the use of conventional iron ore. Conventionally, materials such as cement, which is an output of an emission-intensive industry, would be used where the extraction of iron ore is concerned, causing higher emissions ( Haque, 2022). The use of biomass-based binders in manufacturing carbonaceous pellets lessens the need for mineral resource-based products. According to B1-B5 scenarios, a reduction of MRS by 4.99 % can be seen in substituting 30 % of the sinter with biomass-based pellets. The scenario N1, which uses coke in the BF, has the highest MRS, indicating that biomass-based materials can reduce the reliance on mineral resources compared to mined inputs.

**Figure 3e.**
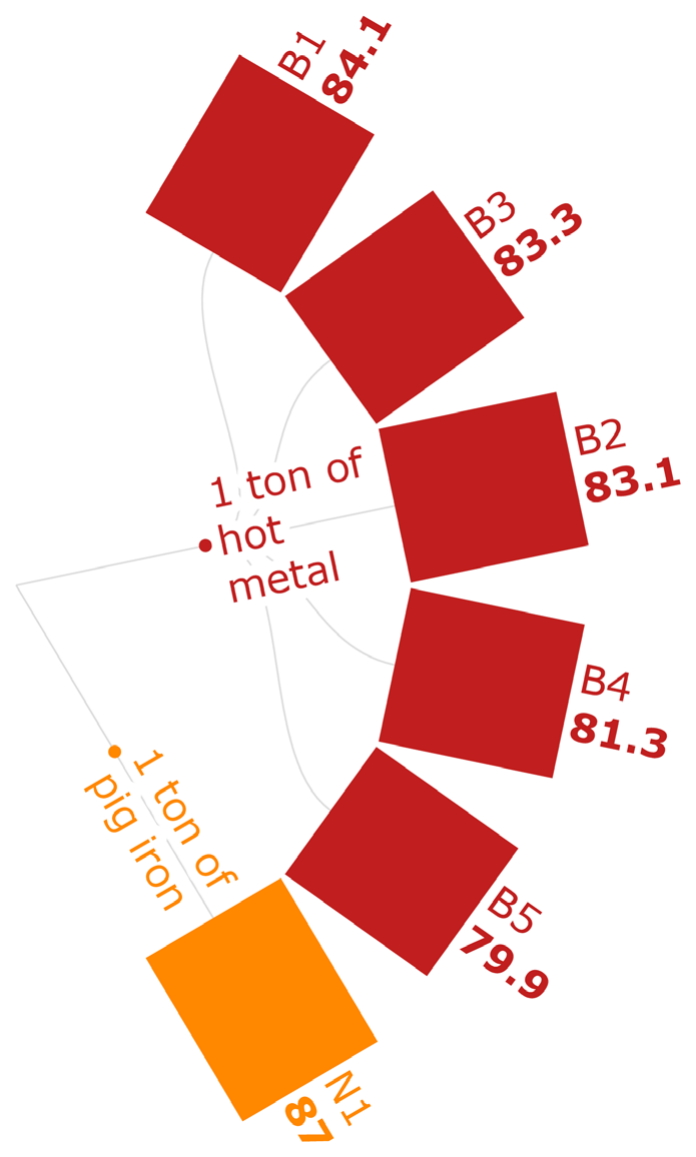
MRS of iron (kg CU eq./FU). Mineral Resource Scarcity of iron (kg CU eq./Functional Unit). Please refer to Table 2 for the descriptions of the scenarios.

FRS can be defined as the depletion of finite non-renewable energy sources due to their extraction and use ( Yu *et al.*, 2023). The FRS values in this analysis range from -485.3 kg oil eq./FU to 577 kg oil eq./FU. Figure 3(f), which discusses fossil resource consumption, attributes the decline in fossil resource usage to the substitution of biomass for PC (B1-B5). Though the FRS has declined with the increase in the quantity of carbonaceous pellets that replace sinter, there is still a reliance on NG as a supporting energy source for the treatment of biomass. It should also be noted that the use of biomass-based pellets contributed to a decline from 494 kg to 454 kg in non-renewable fossil energy consumption. The use of carbon-neutral PKSC (E4), replacing the coal, generated −485.34 kg oil eq./t of iron. The use of PKSC in scenario E2 contributed to an 88.2% FRS reduction and in E3 an 86.1% reduction compared to the baseline. This indicates that FRS will be diminished when coal, NG, and oil are fully or partially replaced using bio-based sources, despite the technical difference of using the PKSC at the discharge end or the feed end of the rotary kiln. Scenarios with PKSC (E2-E4) show up to 88% lower FRS compared to the baseline, as biomass is carbon-neutral and does not require fossil extraction. Scenarios from N1-N4 represent typical cases where top gas and slag are being recycled into the process, thus generating lower FRS values. However, scenario N5 is a prospective analysis of how co-products and blends of inputs coupled with renewable electricity mixes can generate lower FRS values. 

**Figure 3f.**
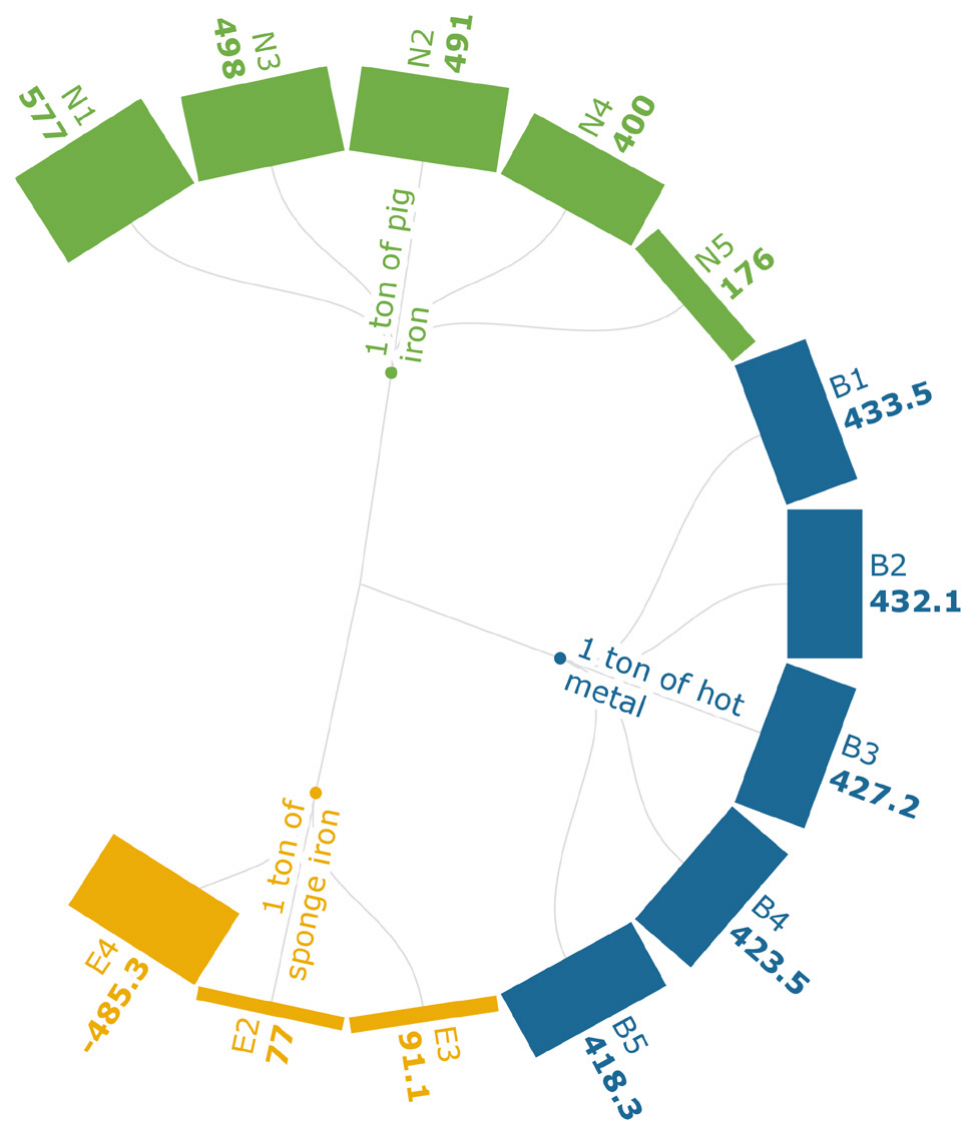
FRS of iron (kg oil eq./FU). Fossil Resource Scarcity of iron (kg oil eq./Functional Unit). Please refer to Table 2 for the descriptions of the scenarios.

## Discussion

The results of this systematic review discuss the use of LCA in the sustainability assessments of hydrogen and/or biomass integrated iron and steel manufacturing scenarios. The technical, spatial scope and impact assessment results were used to compare the available case studies in the literature. Therefore, in this section, we have compiled the findings of hydrogen-based steel LCA studies first, and subsequently the findings of biomass-based LCA studies according to the technical and regional differences in goal and scope definition and impact categories. This section will wrap up with an acknowledgement of the limitations of the current study.

**Figure 4.**
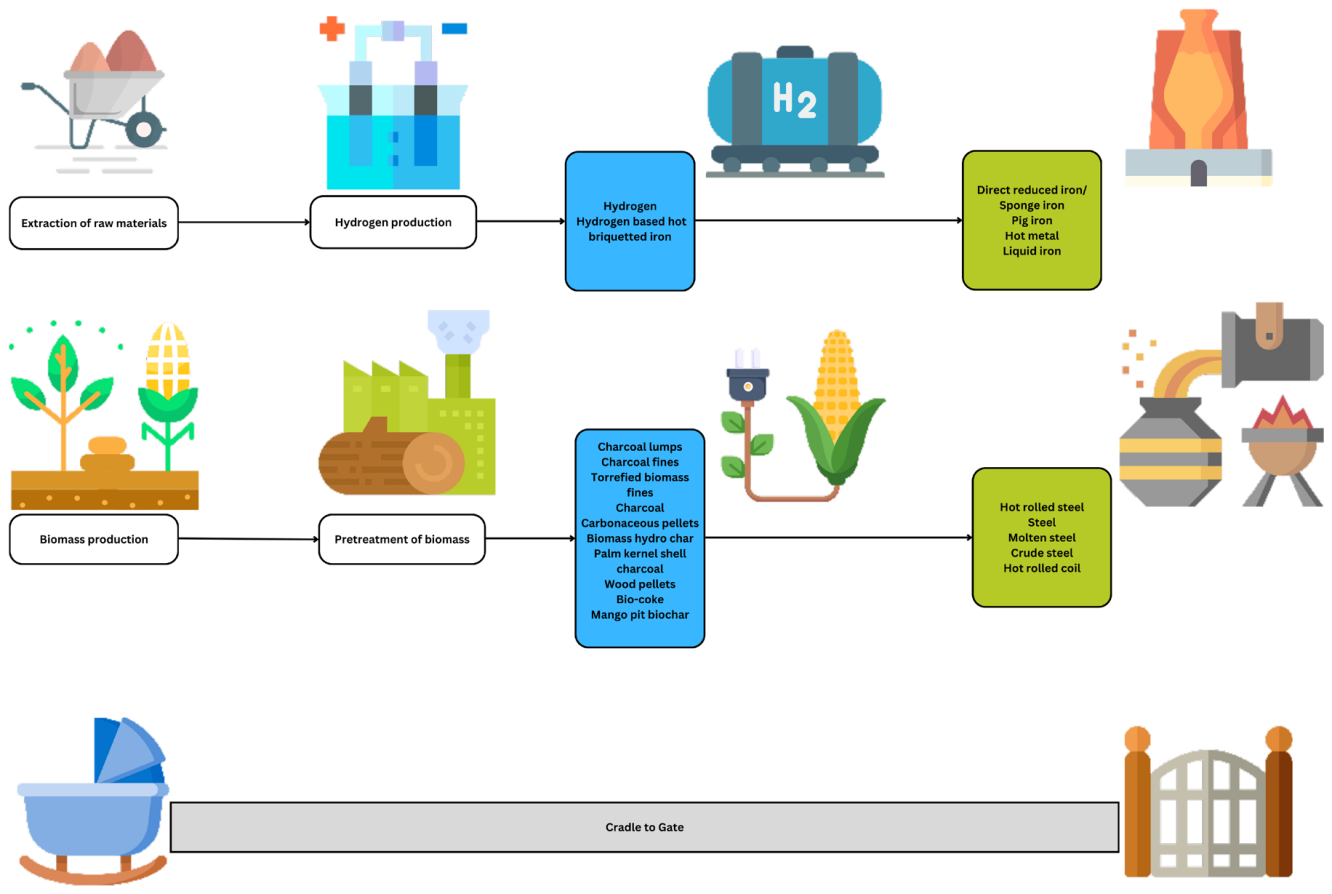
The technological scope of the assessed case studies. The integration methods of hydrogen and/or biomass in iron and/or steel production.

The technical and regional analysis of the hydrogen-based steel LCAs in this review covers multiple technological pathways, such as utilizing hydrogen in the direct reduction process, shaft reduction, BF, EAF, BOF, and hydrogen in the form of HBI. According to Figure 4, the fundamental role of hydrogen in ISI is being a reducing agent that can fully or partially replace fossil-based reducing agents such as natural gas. In the current sample of studies, hydrogen is being used frequently in direct reduction, shaft reduction, or in the blast furnace as a reliable reducing agent. Some studies incorporate hydrogen in the form of inputs. For example, the use of hydrogen in HBI in the BF represents a case where hydrogen is being used in the upstream processes. Either way, hydrogen in the form of a reducing agent or HBI represents higher energy efficiencies, leading to lower emissions in ISI. Additionally, four of the assessed case studies associated with hydrogen integration in the ISI show that the environmental impacts associated with hydrogen-producing methods, such as coal gasification, water electrolysis, bio-syngas, and SCWG, stress the importance of the renewability of the hydrogen-producing source and the use of renewable electricity in producing hydrogen. The regional analysis further indicates how the European ISI is leaning towards embracing the hydrogen economy as the driving force in the steel industry. The Asian case study distribution consists of multiple hydrogen-based alternatives that are mostly at a developing stage, unlike the industrial-scale projects in Europe.

The comparison of impact assessment indicators for the hydrogen-based steel studies scenarios affirms that integrating hydrogen into the ISI will generate environmental benefits. All hydrogen-based steel LCA studies considered were conducted from cradle to gate. It is important that transparent reporting is practiced in hydrogen-based studies in order to ensure fair comparison. For example, the reporting of scenarios accounting for the emissions of the logistics associated with hydrogen transport is important, as the carbon footprint of the transportation of hydrogen varies significantly depending on the mode of transport, storage, and the phases of hydrogen ( Xu *et al.*, 2022). A clear depiction of whether unit processes, such as hydrogen transport, are included in the system boundary of the LCA model is important when determining the benefits of hydrogen integration in ISI. The impact assessment results also indicate that hydrogen provides significant greenhouse gas emission reductions, although this depends on the source of hydrogen and the technical integration. The results further strengthen the fact that hydrogen coupled with renewable electricity can generate higher efficiencies, especially in prospective LCAs.

On the other hand, the use of biomass as an alternative carbon source can be considered as a technically and spatially sensitive research area. Most of the Asian case studies in the current spread of the review evaluate the potential of integrating biomass into the ISI. It is also important to understand that these studies are based on lab-scale experiments and they list multiple potential bottlenecks of scaling up biomass-based steel production, such as the availability and the quality of biomass to fully or partially replace conventional coal. The use of PKSC in India to produce charcoal, wood waste-based biomass in Sweden, and MPB in China are a few derivatives from the analysis, where the form and source of biomass remained vastly different from each other, depending on their region. Regardless of the form of biomass, the case studies weigh heavily towards the practical constraints of a biomass-based ISI. For example, bio-carbon replacement in BF is expected to have practical constraints that hinder its CO _2_ mitigation potential, whereas the fuel injection via the tuyeres is the best way to mitigate CO _2_ emissions in the BF while maintaining the operating efficiency ( Ji *et al*., 2024). This explains the difficulties of adapting the existing technical structures to accommodate biomass-based carbon sources ( Suopajärvi *et al.*, 2017).

The impact assessment of biomass-based steel studies has also indicated the need to establish a standardized method of conducting LCAs for biomass-based studies, as the assumptions and the system boundaries used in calculations can affect the final results of the study. The current sample of studies in this review was all cradle to gate assessments but for example, certain studies did not have data on capital goods and infrastructure construction, which affects the terrestrial habitats, the soil, and land use ( Liang *et al*., 2020). Also, emerging technology integration causes the issue of impact displacement ( Harvey *et al*., 2022). For example, in biomass-based studies, it can be noted that the impact on land and water use change must be assessed before ensuring a continuous supply of biomass feedstock. Land use and water use are two potential indicators that will increase in the case of cultivating biomass using existing land area for industrial purposes. It should also be noted that unless the biomass has been extracted from waste and residues, it cannot necessarily be considered biogenic, as the biomass extracted from harvested sites is either incinerated or disposed of into the atmosphere, with extractable energy present in it ( Wang & Tester, 2023a). Therefore, a set of guidelines is needed on how the credits and the biogenic carbon emissions should be implemented in an LCA software, depending on the replacement ratios of biomass-based materials to fossil-based materials.

A concern that has been addressed in many papers is the lack of a primary data inventory for biomass integration ( Valipour *et al*., 2024). This is especially relevant for the upstream processing of biomass due to the significant amount of indirect and avoided emissions that are not being accounted for in the biomass-based LCAs in usual cases. These emissions are often assumed to be null, which can be theoretically accurate but realistically incorrect ( Nurdiawati *et al*., 2023). For example, the thermochemical upgrading processes of biomass require a significant amount of energy, thus increasing the emissions of biomass use ( Peng *et al*., 2023). Also, the GWI of biomass is strongly sensitive to several upstream processes other than thermal upgrading, such as cultivation practices, transport, and further treatments ( Fan & Friedmann, 2021).

The availability of data also ensures the ability to measure the impact on various impact categories and improves the comprehensiveness of the study ( Suer *et al.*, 2022b). For example, the absence of indicators such as Cumulative Energy Demand (CED) to measure the energy intensity of the integrated ISI and Land Transition (LT) to analyze the impact of biomass cultivation for harvesting, hinders an effective LCA evaluation of the proposed studies. Also, the significance of gathering data from onsite observations has been pointed out several times to increase the reliability of the LCA study. The detailed modeling in simulation software, harnessed together with field data and literature values, with correct assumptions, will benefit the completeness of the life cycle inventory immensely in biomass-based LCAs. Additionally, several studies suggest the use of Prospective LCA and Life Cycle Sustainability Assessment (LCSA) to assess the impact of decarbonizing energy systems and assess the social and economic aspects of LCA to have better insights into these studies ( Suer *et al*., 2022a).

Finally, the limitations of our review can be acknowledged. First, only peer-reviewed articles were considered for the review. Other sources, such as grey literature, were excluded, although they may still contain valid and useful findings. The search query for this systematic review was established using the keywords that best represented the research questions of our study. Therefore, the search query only used LCA in its search, though it could be extended further with other relevant keywords such as footprint. The search string itself generated a considerable number of results initially, but several studies had to be excluded due to the multi-disciplinary nature of the studies presented, as most of the process engineering publications did not use LCA for emission computations, thus disqualifying them for the final synthesis. Several limitations were inherent to the LCA methodology when interpreting the results, such as uncertainties associated with the LCA inventory in terms of technological variations, data quality, and the temporal scope of the data. This review also does not include social or economic dimensions, and future research will benefit from an integrated socio-economic environmental perspective on hydrogen and/or biomass integrations in steel. 

## Conclusion

This research explores the life cycle contributions of several hydrogen and/or biomass integrations in ISI. The novelty of the work lies in its attempt to aggregate current studies and analyze those depending on their goal and scope definition, and impact indicators to assist future research on hydrogen and/or biomass in ISI. The technical and regional analysis of the case studies indicated that various integrating approaches exist, depending on key geographical features, and that they depend heavily on resource availability and access to renewable energy systems. The comparison of impact indicators showed that the use of hydrogen and/or biomass can generally create a positive impact on environmental preservation. It is important that these numbers are interpreted with caution, as the values may vary widely depending on the technical integration of the scenarios. Furthermore, this review identified that there was also a notable absence of an agreed-upon method of LCA computing for the quantification of environmental impacts associated with biomass integration, leading to LCA reporting discrepancies. Thus, it is important that transparent LCA reporting guidelines are being practiced while the LCA methodology itself is modified and standardized to address the technical and spatially sensitive research. Overall, this work further strengthens the idea that emerging technologies require sustainability assessment techniques such as LCA, applied on lab and commercial scales to ascertain their sustainability before a major roll-out of infrastructure on an industrial scale.

## Ethics and consent

Ethical approval and consent were not required for this systematic review.

## Data Availability

Zenodo: Extended data for 'Hydrogen and biomass-based carbon source integration for iron and steel manufacturing: A systematic review of Life Cycle Assessment studies'. https://zenodo.org/records/18036212 Zenodo: PRISMA 2020 checklist and flow diagram for ‘Hydrogen and biomass-based carbon source integration for iron and steel manufacturing: A systematic review of Life Cycle Assessment studies’. https://zenodo.org/records/18036134 Data are available under the terms of the Creative Commons Attribution 4.0 International license (CC-BY 4.0) ( https://creativecommons.org/licenses/by/4.0/).

## References

[ref-1] Arvidsson R Svanström M Harvey S : Life-cycle impact assessment methods for physical energy scarcity: considerations and suggestions. *Int J Life Cycle Assess.* 2021;26(12):2339–2354. 10.1007/s11367-021-02004-x

[ref-107] Atrens A Liu Q Tapia-Bastidas C : Influence of hydrogen on steel components for clean energy. *Corros Mater Degrad.* 2020;1(1):3–26. 10.3390/cmd1010002

[ref-2] Azevedo LB Roy PO Verones F : 7. Terrestrial acidification.2012. Reference Source

[ref-3] Bailera M Lisbona P Peña B : A review on CO _2_ mitigation in the Iron and Steel industry through Power to X processes. *J CO2 Util.* 2021;46: 101456. 10.1016/J.JCOU.2021.101456

[ref-109] Baitz M : Responsibility in life cycle assessment practice. *Int J Life Cycle Assess.* 2019;24(2):179–180. 10.1007/s11367-018-1558-1

[ref-4] Bataille C Åhman M Neuhoff K : A review of technology and policy deep decarbonization pathway options for making energy-intensive industry production consistent with the Paris Agreement. *J Clean Prod.* 2018;187:960–973. 10.1016/J.JCLEPRO.2018.03.107

[ref-5] Bersenev IS Vokhmyakova IS Borodin AV : Prediction of the quality of Hot Briquetted Iron (HBI) based on data on the material composition of pellets. *Steel Trans.* 2022;52(7):673–676. 10.3103/S0967091222070038

[ref-110] Bhaskar A Abhishek R Assadi M : Decarbonizing primary steel production : Techno-economic assessment of a hydrogen based green steel production plant in Norway. *J Clean Prod.* 2022;350: 131339. 10.1016/j.jclepro.2022.131339

[ref-6] Bilby BA Hewitt J : Hydrogen in steel—the stability of micro-cracks. *Acta Metallurgica.* 1962;10(6):587–600. 10.1016/0001-6160(62)90048-2

[ref-7] Bolívar Caballero JJ Zaini IN Yang W : Reforming processes for syngas production: a mini-review on the current status, challenges, and prospects for biomass conversion to fuels. *Appl Energy Combust Sci.* 2022;10: 100064. 10.1016/J.JAECS.2022.100064

[ref-8] Boretti A : Production of hydrogen for export from wind and solar energy, natural gas, and coal in Australia. *Int J Hydrogen Energy.* 2020;45(7):3899–3904. 10.1016/J.IJHYDENE.2019.12.080

[ref-9] Burchart-Korol D : Life cycle assessment of steel production in Poland: a case study. *J Clean Prod.* 2013;54:235–243. 10.1016/J.JCLEPRO.2013.04.031

[ref-10] Cao C : Sustainability and life assessment of high strength natural fibre composites in construction. *Advanced High Strength Natural Fibre Composites in Construction.* 2017;529–544. 10.1016/B978-0-08-100411-1.00021-2

[ref-11] Cardarelli A Barbanera M : Substitution of fossil coal with hydrochar from agricultural waste in the Electric Arc Furnace steel industry: a comprehensive life cycle analysis. *Energies.* 2023;16(15):5686. 10.3390/en16155686

[ref-12] Chan Y Petithuguenin L Fleiter T : Industrial innovation: pathways to deep decarbonisation of industry Part 1: technology analysis a report submitted by ICF Consulting Services Limited and Fraunhofer ISI to the European Commission, DG climate action prepared by checked by publication date. 2019. Reference Source

[ref-13] Cooper J Hawkes A : Life cycle environmental trade-off of decarbonising UK industrial clusters — a cradle to gate approach. *Sci Total Environ.* 2024;954: 176101. 10.1016/j.scitotenv.2024.176101 39265687

[ref-122] Dodd N Cordella M Traverso M : Level(s) – A common EU framework of core sustainability indicators for office and residential buildings: Parts 1 and 2: Introduction to Level(s) and how it works (Beta v1.0).2017. Reference Source

[ref-14] Doranehgard MH Samadyar H Mesbah M : High-purity hydrogen production with in situ CO _2_ capture based on biomass gasification. *Fuel.* 2017;202:29–35. 10.1016/J.FUEL.2017.04.014

[ref-15] Drielsma JA Russell-Vaccari AJ Drnek T : Mineral resources in life cycle impact assessment—defining the path forward. *Int J Life Cycle Assess.* 2016;21(1):85–105. 10.1007/S11367-015-0991-7

[ref-16] Durga S Speizer S Edmonds J : The role of the iron and steel sector in achieving net zero U.S. CO _2_ emissions by 2050. *Energy Clim Chang.* 2024;5: 100152. 10.1016/J.EGYCC.2024.100152

[ref-17] Edenhofer O Sokona Y Minx JC : Climate change 2014 mitigation of climate change summary for policymakers technical summary Part of the Working Group III contribution to the Fifth assessment report of the intergovernmental panel on climate change. Edited by.2015. 10.1017/CBO9781107415416

[ref-18] Egerer J Farhang-Damghani N Grimm V : The industry transformation from fossil fuels to hydrogen will reorganize value chains: big picture and case studies for Germany. *Appl Energy.* 2024;358: 122485. 10.1016/J.APENERGY.2023.122485

[ref-20] European Commission: International Reference Life Cycle Data system (ILCD) handbook - general guide for Life Cycle Assessment - provisions and action steps.Publications Office of the European Union: Luxembourg,2010;150. Reference Source

[ref-21] Fan Z Friedmann SJ : Low-carbon production of iron and steel: technology options, economic assessment, and policy. *Joule.* 2021;5(4):829–862. 10.1016/J.JOULE.2021.02.018

[ref-22] Farhana Diyana Mohd Yunos N Nadhirah Ismail A Farhana Yunos NM : Transforming waste materials as resources for EAF steelmaking. *Int J Mater Eng.* 2015;2014(5):167–170. Reference Source

[ref-23] Fick G Mirgaux O Neau P : Using biomass for pig iron production: a technical, environmental and economical assessment. *Waste Biomass Valor.* 2014;5(1):43–55. 10.1007/s12649-013-9223-1

[ref-24] Finkbeiner M Inaba A Tan RBH : The new international standards for life cycle assessment: ISO 14040 and ISO 14044. *Int J Life Cycle Assess.* 2006;11(2):80–85. 10.1065/lca2006.02.002

[ref-25] Finnveden G Hauschild MZ Ekvall T : Recent developments in life cycle assessment. *J Environ Manage.* 2009;91(1):1–21. 10.1016/J.JENVMAN.2009.06.018 19716647

[ref-26] Furberg A Arvidsson R Molander S : A practice-based framework for defining functional units in comparative life cycle assessments of materials. *J Ind Ecol.* 2022;26(3):718–730. 10.1111/jiec.13218

[ref-27] Gaurav G Bihari Singh A Mistry S : Recent progress of scientific research on life cycle assessment. *Mater Today Proc.* 2021;47(11):3161–3170. 10.1016/j.matpr.2021.06.208

[ref-28] Gielen D Taibi E Miranda R : HYDROGEN: A RENEWABLE ENERGY PERSPECTIVE. 2019. Reference Source

[ref-200] Guinee JB : Handbook on life cycle assessment operational guide to the ISO standards. *Int J Life Cycle Assess.* 2002;7:311–313. 10.1007/BF02978897

[ref-29] Hagemann N Spokas K Schmidt HP : Activated carbon, biochar and charcoal: linkages and synergies across pyrogenic carbon’s *ABC*s. *Water.* 2018;10(2):182. 10.3390/w10020182

[ref-30] Haque N : Life cycle assessment of iron ore mining and processing. *Iro: Mineralogy, Processing and Environmental Sustainability.* 2022;691–710. 10.1016/B978-0-12-820226-5.00007-0

[ref-31] Harvey JP Courchesne W Vo MD : Greener reactants, renewable energies and environmental impact mitigation strategies in pyrometallurgical processes: a review. *MRS Energy Sustain.* 2022;9(2):212–247. 10.1557/s43581-022-00042-y 36569468 PMC9766879

[ref-32] Hou Q Mao G Zhao L : Mapping the scientific research on life cycle assessment: a bibliometric analysis. *Int J Life Cycle Assess.* 2015;20(4):541–555. 10.1007/s11367-015-0846-2

[ref-33] Huang Y Chen J Liu Y : Life cycle assessment of a process integrating supercritical water gasification with direct reduced iron production. *J Clean Prod.* 2024;483: 144250. 10.1016/j.jclepro.2024.144250

[ref-62] International Standard Organization: ISO 14040: environmental management-Life cycle assessment-Principles and framework.1997. Reference Source

[ref-34] IPCC: Mitigation pathways compatible with 1.5°C in the context of sustainable development. *Global Warming of 1.5°C.* 2022;93–174. 10.1017/9781009157940.004

[ref-35] Islam A Islam T Mahmud H : Accelerating the green hydrogen revolution: a comprehensive analysis of technological advancements and policy interventions. *Int J Hydrogen Energy.* 2024;67:458–486. 10.1016/J.IJHYDENE.2024.04.142

[ref-36] Jahanshahi S Mathieson JG Somerville MA : Development of low-emission integrated steelmaking process. *J Sustain Metall.* 2015;1(1):94–114. 10.1007/s40831-015-0008-6

[ref-37] Ji Z Yu D Fan X : Resourceful utilization of combustible solid wastes throughout steelmaking processes: recent progress and prospects. *J Clean Prod.* Elsevier Ltd,2024;449: 141696. 10.1016/j.jclepro.2024.141696

[ref-39] Katrak FE : Iron and steel, future of. *Encyclopedia of Materials: Science and Technology.* 2001;4292–4295. 10.1016/B0-08-043152-6/00753-1

[ref-40] Khasraw D Martin C Herbert J : A comprehensive literature review of biomass characterisation and application for iron and steelmaking processes. *Fuel.* 2024;368: 131459. 10.1016/J.FUEL.2024.131459

[ref-41] Khatri P Pandit AB : Systematic review of life cycle assessments applied to sugarcane bagasse utilization alternatives. *Biomass Bioenergy.* 2022;158: 106365. 10.1016/J.BIOMBIOE.2022.106365

[ref-42] Leão AS Medeiros DL Santiago MA : Rigorous environmental and energy life cycle assessment of blast furnace pig iron in Brazil: the role of carbon and iron sources, and co-product utilization. *Sustainable Materials and Technologies.* 2023;36: e00607. 10.1016/j.susmat.2023.e00607

[ref-43] Lehmann J Joseph S : Fundamentals of biochar production. *Biochar for Environmental Management.* 2015;39–61.

[ref-46] Li J Cheng W : Comparative life cycle energy consumption, carbon emissions and economic costs of hydrogen production from coke oven gas and coal gasification. *Int J Hydrogen Energy.* 2020;45(51):27979–27993. 10.1016/J.IJHYDENE.2020.07.079

[ref-44] Li F Chu M Tang J : Thermodynamic performance analysis and environmental impact assessment of an integrated system for hydrogen generation and steelmaking. *Energy.* 2022a;241: 122922. 10.1016/j.energy.2021.122922

[ref-45] Li F Chu M Tang J : Quantifying the energy saving potential and environmental benefit of hydrogen-based steelmaking process: status and future prospect. *Appl Therm Eng.* 2022b;211: 118489. 10.1016/j.applthermaleng.2022.118489

[ref-47] Liang T Wang S Lu C : Environmental impact evaluation of an iron and steel plant in China: normalized data and direct/indirect contribution. *J Clean Prod.* 2020;264: 121697. 10.1016/j.jclepro.2020.121697

[ref-48] Liang W Wang G Xu R : Life cycle assessment of blast furnace ironmaking processes: a comparison of fossil fuels and biomass hydrochar applications. *Fuel.* 2023;345: 128138. 10.1016/j.fuel.2023.128138

[ref-49] Lin YP Wang WH Pan SY : Environmental impacts and benefits of organic Rankine cycle power generation technology and wood pellet fuel exemplified by electric arc furnace steel industry. *Appl Eng.* 2016;183:369–379. 10.1016/j.apenergy.2016.08.183

[ref-50] Liu W Zuo H Wang J : The production and application of hydrogen in steel industry. *Int J Hydrogen Energy.* 2021;46(17):10548–10569. 10.1016/J.IJHYDENE.2020.12.123

[ref-51] Lu C Zhang D Ren J : Life cycle assessment of carbonaceous pellets used in blast furnaces in the context of “double carbon”. *Sci Total Environ.* 2024;935: 173274. 10.1016/j.scitotenv.2024.173274 38754508

[ref-116] MacFarlane A Russell-Rose T Shokraneh F : Search strategy formulation for systematic reviews: issues, challenges and opportunities. *Intell Syst Appl.* 2022;15: 200091. 10.1016/j.iswa.2022.200091

[ref-52] Mandova H : Assessment of bioenergy as a CO _2_ emission reduction strategy for European iron and steelmaking.2019. Reference Source

[ref-117] Meho LI Yang K : A new era in citation and bibliometric analyses: Web of Science, Scopus, and Google Scholar.arXiv:cs/0612132.2006;49. 10.48550/arXiv.cs/0612132

[ref-53] Moher D Liberati A Tetzlaff J : Preferred reporting items for systematic reviews and meta-analyses: the PRISMA statement. *BMJ.* 2009;339(7716): b2535. 10.1136/bmj.b2535 19622551 PMC2714657

[ref-54] Mongeon P Paul-Hus A : The journal coverage of Web of Science and Scopus: a comparative analysis. *Scientometrics.* 2016;106(1):213–228. 10.1007/s11192-015-1765-5

[ref-55] Mousa E Wang C Riesbeck J : Biomass applications in iron and steel industry: an overview of challenges and opportunities. *Renew Sustain Energy Rev.* 2016;65:1247–1266. 10.1016/j.rser.2016.07.061

[ref-56] Muritala IK Guban D Roeb M : High temperature production of hydrogen: assessment of non-renewable resources technologies and emerging trends. *Int J Hydrogen Energy.* 2020;45(49):26022–26035. 10.1016/j.ijhydene.2019.08.154

[ref-57] Nassaralla CL : Iron production. *Encyclopedia of Materials: Science and Technology.* 2001;4296–4301. 10.1016/B0-08-043152-6/00754-3

[ref-58] Neuwirth M Fleiter T Manz P : The future potential hydrogen demand in energy-intensive industries - a site-specific approach applied to Germany. *Energy Convers Manag.* 2022;252: 115052. 10.1016/j.enconman.2021.115052

[ref-59] Nurdiawati A Zaini IN Wei W : Towards fossil-free steel: life cycle assessment of biosyngas-based Direct Reduced Iron (DRI) production process. *J Clean Prod.* 2023;393: 136262. 10.1016/j.jclepro.2023.136262

[ref-60] Nwachukwu CM Olofsson E Lundmark R : Evaluating fuel switching options in the Swedish Iron and Steel Industry under increased competition for forest biomass. *Appl Energy.* 2022;324: 119878. 10.1016/j.apenergy.2022.119878

[ref-61] Nwachukwu CM Wang C Wetterlund E : Exploring the role of forest biomass in abating fossil CO _2_ emissions in the iron and steel industry – the case of Sweden. *Appl Energy.* 2021;288: 116558. 10.1016/j.apenergy.2021.116558

[ref-108] Oriani RA : The diffusion and trapping of hydrogen in steel. *Acta Metallurgica.* 1970;18(1):147–157. 10.1016/0001-6160(70)90078-7

[ref-63] Peng X Jiang Y Chen Z : Recycling municipal, agricultural and industrial waste into energy, fertilizers, food and construction materials, and economic feasibility: a review. *Environ Chem Lett.* 2023;21(2):765–801. 10.1007/s10311-022-01551-5

[ref-64] Petrescu L Chisalita DA Cormos CC : Life cycle assessment of SEWGS technology applied to integrated steel plants. *Sustainability.* 2019;11(7): 1825. 10.3390/su11071825

[ref-65] Pitsch H : The transition to sustainable combustion: hydrogen- and carbon-based future fuels and methods for dealing with their challenges. *Proc Combust Inst.* 2024;40(1–4): 105638. 10.1016/j.proci.2024.105638

[ref-121] Ponomarenko T Nevskaya M Jonek-Kowalska I : Mineral resource depletion assessment: alternatives, problems, results. *Sustainability.* 2021;13(2):862. 10.3390/su13020862

[ref-113] Pranckutė R : Web of Science (WoS) and Scopus: the titans of bibliographic information in today’s academic world. *MDPI.* 2021;9(1):12. 10.3390/publications9010012

[ref-66] Quader MA Ahmed S Ghazilla RAR : A comprehensive review on energy efficient CO _2_ breakthrough technologies for sustainable green iron and steel manufacturing. *Renew Sustain Energy Rev.* 2015;50:594–614. 10.1016/j.rser.2015.05.026

[ref-67] Ranzani Da Costa A Wagner D Patisson F : Modelling a new, low CO _2_ emissions, hydrogen steelmaking process. *J Clean Prod.* 2013;46:27–35. 10.1016/j.jclepro.2012.07.045

[ref-68] Ren L Zhou S Ou X : The carbon reduction potential of hydrogen in the low carbon transition of the iron and steel industry: the case of China. *Renew Sustain Energy Rev.* 2023;171: 113026. 10.1016/j.rser.2022.113026

[ref-69] Richardson DB Blanzymski TZ Gregory EN : Manufacturing methods. *Mech Eng Ref Book.* 1994;16–1. 10.1016/B978-0-7506-1195-4.50020-8

[ref-70] Rodríguez-Narvaez OM Medina-Orendain DA Mendez-Alvarado LN : Functionalized green carbon-based nanomaterial for environmental application. *Sust Nanotechnol Environ Remediat.* 2022;347–382. 10.1016/B978-0-12-824547-7.00005-9

[ref-71] Samarasekera IV : Hot rolling. *Encyclopedia of Materials: Science and Technology.* 2001;3836–3843. 10.1016/B0-08-043152-6/00683-5

[ref-112] Sambandam M Nurni VN Jayaraj SP : Sustainable production of steel–carbon neutrality and low life cycle emission. * J Indian Inst Sci.* 2022;102:117–126. 10.1007/s41745-021-00285-7

[ref-72] Sangkham S Phairuang W Sherchan SP : An update on adverse health effects from exposure to PM _2.5_. *Environ Adv.* 2024;18: 100603. 10.1016/j.envadv.2024.100603

[ref-73] Schinkel U Becker N Trapp M : Assessing the contribution of innovative technologies to sustainable development for planning and decision-making processes: a set of indicators to describe the performance of sustainable urban infrastructures (ISI). *Sustainability.* 2022;14(4): 1966. 10.3390/su14041966

[ref-74] Sekiguchi N : Steel trade structure and the balance of steelmaking technologies in non-OECD countries: the implications for catch-up path. *Miner Econ.* 2019;32(3):257–285. 10.1007/s13563-018-0163-x

[ref-75] Shahabuddin M Alam MT Krishna BB : A review on the production of renewable aviation fuels from the gasification of biomass and residual wastes. *Bioresour Technol.* 2020;312: 123596. 10.1016/j.biortech.2020.123596 32507633 PMC7255753

[ref-76] Song B Lin R Lam CH : Recent advances and challenges of inter-disciplinary biomass valorization by integrating hydrothermal and biological techniques. *Renew Sustain Energy Rev.* 2021;135: 110370. 10.1016/J.RSER.2020.110370

[ref-77] Souza AM Ribeiro RV Oliveira LD : How upstream methane emissions can impact cost and emissions of steelmaking routes? *J Mater Res Technol.* 2023;24:7153–7161. 10.1016/j.jmrt.2023.04.238

[ref-78] Su Z Anderson CG Long H : Review of Life Cycle Assessments for steel and environmental analysis of future steel production scenarios. *Sustainability.* 2022;14(21): 14131.

[ref-79] Suer J Traverso M Ahrenhold F : Carbon footprint of scenarios towards climate-neutral steel according to ISO 14067. * J Clean Prod.* 2021;318: 128588. 10.1016/j.jclepro.2021.128588

[ref-80] Suer J Traverso M Jäger N : Carbon footprint assessment of hydrogen and steel. *Energies.* 2022a;15(24): 9468. 10.3390/en15249468

[ref-81] Suer J Traverso M Jäger N : Review of Life Cycle Assessments for steel and environmental analysis of future steel production scenarios. *Sustainability (Switzerland).* MDPI, November 1,2022b;14: 14131. 10.3390/su142114131

[ref-82] Suopajärvi H Kemppainen A Haapakangas J : Extensive review of the opportunities to use biomass-based fuels in iron and steelmaking processes. *J Clean Prod.* 2017;148:709–734. 10.1016/J.JCLEPRO.2017.02.029

[ref-115] Suopajärvi H Umeki K Mousa E : Use of biomass in integrated steelmaking – Status quo, future needs and comparison to other low-CO _2_ steel production technologies. *Applied Energy.* 2018;213:384–407. 10.1016/j.apenergy.2018.01.060

[ref-83] Swennenhuis F de Gooyert V de Coninck H : Towards a CO _2_-neutral steel industry: justice aspects of CO _2_ capture and storage, biomass- and green hydrogen-based emission reductions. *Energy Res Soc Sci.* 2022;88: 102598. 10.1016/J.ERSS.2022.102598

[ref-84] Tait MW Cheung WM : A comparative cradle-to-gate life cycle assessment of three concrete mix designs. *Int J Life Cycle Assess.* 2016;21(6):847–860. 10.1007/S11367-016-1045-5

[ref-85] Tian S Jiang J Zhang Z : Inherent potential of steelmaking to contribute to decarbonisation targets via industrial Carbon Capture and Storage. *Nat Commun.* 2018;9(1): 4422. 10.1038/s41467-018-06886-8 30356137 PMC6200798

[ref-87] Valipour M Mafakheri F Gagnon B : Integrating bio-hubs in biomass supply chains: insights from a systematic literature review. *J Clean Prod.* 2024;467: 142930. 10.1016/J.JCLEPRO.2024.142930

[ref-86] Vigneswaran VS Gowd SC Rajendran K : Pathways for decarbonizing the Sponge Iron industries: effect of energy balance and impact assessment. *J Clean Prod.* 2024;450: 141962. 10.1016/j.jclepro.2024.141962

[ref-88] Vögele S Grajewski M Govorukha K : Challenges for the European steel industry: analysis, possible consequences and impacts on sustainable development. *Appl Energy.* 2020a;264: 114633. 10.1016/J.APENERGY.2020.114633

[ref-89] Vögele S Grajewski M Govorukha K : Challenges for the European steel industry: analysis, possible consequences and impacts on sustainable development. *Appl Energy.* 2020b;264: 114633. 10.1016/J.APENERGY.2020.114633

[ref-90] Wang K Tester JW : Sustainable management of unavoidable biomass wastes. *Green Energy and Resources.* 2023a;1(1): 100005. 10.1016/J.GERR.2023.100005

[ref-91] Wang Y Wu JJ : Thermochemical conversion of biomass: potential future prospects. *Renew Sustain Energy Rev.* 2023b;187: 113754. 10.1016/j.rser.2023.113754

[ref-92] Watari T McLellan B : Global demand for green hydrogen-based steel: insights from 28 scenarios. *Int J Hydrogen Energ.* 2024;79:630–635. 10.1016/j.ijhydene.2024.06.423

[ref-93] Weckenborg C Graupner Y Spengler TS : Prospective assessment of transformation pathways toward low-carbon steelmaking: evaluating economic and climate impacts in Germany. *Resour Conserv Recycl.* 2024;203: 107434. 10.1016/j.resconrec.2024.107434

[ref-94] Wei R Meng K Long H : Biomass metallurgy: a sustainable and green path to a carbon-neutral metallurgical industry. *Renew Sustain Energy Rev.* 2024;199: 114475. 10.1016/j.rser.2024.114475

[ref-95] Xi H Wu X Chen X : Artificial Intelligent based energy scheduling of steel mill gas utilization system towards carbon neutrality. *Appl Energ.* 2021;295: 117069. 10.1016/j.apenergy.2021.117069

[ref-96] Xu B Lin B : Regional differences in the CO _2_ emissions of China’s iron and steel industry: regional heterogeneity. *Energy Policy.* 2016;88:422–434. 10.1016/j.enpol.2015.11.001

[ref-97] Xu X Zhou Q Yu D : The future of hydrogen energy: bio-hydrogen production technology. *Int J Hydrogen Energ.* 2022;47(79):33677–33698. 10.1016/j.ijhydene.2022.07.261

[ref-98] Xylia M Silveira S Duerinck J : Weighing regional scrap availability in global pathways for steel production processes. *Energ Effic.* 2018;11(5):1135–1159. 10.1007/s12053-017-9583-7

[ref-99] Yang M Peng Q Cao G : Feasibility analysis and environmental impact evaluation of biochar derived from mango pit for Blast Furnace injection. *Chem Eng J.* 2024;487: 150451. 10.1016/j.cej.2024.150451

[ref-120] Yu M Wenhui X Anser MK : Navigating the global mineral market: a study of resource wealth and the energy transition. *Resources Policy.* 2023;82: 103500. 10.1016/j.resourpol.2023.103500

[ref-100] Zakaria MR Ahmad Farid MA Andou Y : Production of biochar and activated carbon from oil palm biomass: current status, prospects, and challenges. *Ind Crop Prod.* 2023;199: 116767. 10.1016/j.indcrop.2023.116767

[ref-119] Zama N Kirkman K Mkhize N : Soil acidification in nutrient-enriched soils reduces the growth, nutrient concentrations, and nitrogen-use efficiencies of *Vachellia sieberiana* (DC.) Kyal. & Boatwr saplings. *Plants.* 2022;11(24):3564. 10.3390/plants11243564 36559678 PMC9781205

[ref-101] Zang G Sun P Elgowainy A : Cost and life cycle analysis for deep CO _2_ emissions reduction for steel making: direct reduced iron technologies. *Steel Res Int.* 2023;94(6): 2200297. 10.1002/srin.202200297

[ref-118] Zhang J Fu H Liu Y : Review on biomass metallurgy: pretreatment technology, metallurgical mechanism and process design. *International Journal of Minerals, Metallurgy and Materials.* 2022;29:1133–1149. 10.1007/s12613-022-2501-9

[ref-103] Zhang X Jiao K Zhang J : A review on low carbon emissions projects of steel industry in the world. *J Clean Prod.* 2021a;306: 127259. 10.1016/j.jclepro.2021.127259

[ref-102] Zhang J Shen H Chen Y : Iron and steel industry emissions: a global analysis of trends and drivers. *Environ Sci Technol.* 2023;57(43):16477–16488. 10.1021/acs.est.3c05474 37867432 PMC10621597

[ref-105] Zhao P Dong PL : Carbon emission cannot be ignored in future of Chinese steel industry. *Kang T’ieh/Iron and Steel.* 2018;53(8):1–7. 10.13228/j.boyuan.issn0449-749x.20180081

[ref-106] Zhou P Zhang R Liang M : Fault identification for quality monitoring of molten iron in Blast Furnace ironmaking based on KPLS with improved contribution rate. *Control Eng Pract.* 2020;97: 104354. 10.1016/j.conengprac.2020.104354

