# Supplementary material for: Hydrogen and biomass-based carbon source integration for iron and steel manufacturing: A systematic review of Life Cycle Assessment studies
Source: Open Res Eur. 2026 Feb 3;5:196. Originally published 2025 Jul 25. [Version 2] doi: 10.12688/openreseurope.20725.2 (PMC12936486; doi:10.12688/openreseurope.20725.2)
Supplement: Peer review history

# Reviewer report: Reviewer response for version 2

**DOI:** 10.21956/openreseurope.24452.r69206

**Published:** 2026-02-25

**Authors:** Heming Wang

**Affiliations:** 1 Northeastern University, Shenyang, China

This is a well-written and logically structured report that addresses a highly relevant topic, the decarbonization of the iron and steel industry. The writing is rigorous and the results are solid. Overall, the premise of the study is significant, and the underlying framework promises to offer valuable, practical insights into the policy design for low-carbon transition of the sector.

 I have one suggestion: while the introduction discusses hydrogen and biomass extensively, it currently treats them as parallel, somewhat disconnected alternatives. The authors need to explicitly justify why these two resources are being evaluated together in a single review rather than in separate reports. What is the underlying correlation between them? Adding a brief discussion on how these two pathways intersect will fully justify the combined scope and significantly strengthen the rationale of the paper.

Are the rationale for, and objectives of, the Systematic Review clearly stated?

Yes

Is the statistical analysis and its interpretation appropriate?

Yes

Are sufficient details of the methods and analysis provided to allow replication by others?

Yes

Are the conclusions drawn adequately supported by the results presented in the review?

Yes

Reviewer Expertise:

Circular Economy, Material Flow Analysis, Sustainability

I confirm that I have read this submission and believe that I have an appropriate level of expertise to confirm that it is of an acceptable scientific standard.

# Reviewer report: Reviewer response for version 2

**DOI:** 10.21956/openreseurope.24452.r69069

**Published:** 2026-02-20

**Authors:** Patcharin Racho

**Affiliations:** 1 Suranaree University of Technology, Nakhon Ratchasima, Thailand

The revised manuscript has been carefully improved in response to the previous comments. The authors have addressed the major concerns in a satisfactory manner, and the clarity and coherence of the paper have been strengthened. The methodological description is now clearer, and the discussion better reflects the scope and limitations of the review.

 Overall, I am satisfied with the revisions made by the authors. The manuscript is now suitable for indexing in its current form.

Are the rationale for, and objectives of, the Systematic Review clearly stated?

Partly

Is the statistical analysis and its interpretation appropriate?

Not applicable

Are sufficient details of the methods and analysis provided to allow replication by others?

Partly

Are the conclusions drawn adequately supported by the results presented in the review?

Partly

Reviewer Expertise:

Environmental engineering; wastewater treatment; anaerobic digestion and bioenergy; life cycle assessment (LCA) of waste-to-energy and industrial processes; circular economy and sustainability assessment of resource recovery systems.

I confirm that I have read this submission and believe that I have an appropriate level of expertise to confirm that it is of an acceptable scientific standard.

# Reviewer report: Reviewer response for version 1

**DOI:** 10.21956/openreseurope.22418.r60489

**Published:** 2025-09-27

**Authors:** Patcharin Racho

**Affiliations:** 1 Suranaree University of Technology, Nakhon Ratchasima, Thailand

**Reviewer’s Comments for Authors**

 Your manuscript addresses an important and timely topic: decarbonisation of the iron and steel industry through hydrogen and biomass integration, assessed via Life Cycle Assessment (LCA). The compilation of 21 studies provides a potentially valuable foundation. However, in its current form the manuscript resembles an annotated bibliography rather than a systematic and critical review. Major revisions are required before the paper can be considered for indexing.

 Major Issues

1. **Conceptual framing and research gap**
  - The introduction does not clearly articulate the unique contribution of this review. Existing review papers on LCA and decarbonisation of steel should be discussed, and the specific research gap (hydrogen + biomass integration in LCA) must be sharpened.
  - The research questions need refinement to highlight methodological drivers (e.g. system boundaries, electricity mix assumptions, biogenic carbon accounting) that explain outcome variability.
2. **Methodological rigour and transparency**
  - Although PRISMA is cited, the process is applied superficially. The search strategy is narrow, database selection not justified, and grey literature is excluded without discussion.
  - Screening procedures are unclear (single vs. multiple reviewers, conflict resolution).
  - The most critical omission is the absence of a **quality assessment** of the 21 included studies. Without this, comparisons of results are unreliable, since high- and low-quality LCAs are treated equally. A structured quality appraisal framework (e.g. ISO 14040/14044 criteria, data source reliability, functional units, allocation, uncertainty analysis) is essential.
3. **Synthesis of findings**
  - Results are descriptive, structured by geography, and lack analytical integration. Figures 3a–3f summarise data but do not explain variability (e.g. why GWI ranges from −845 to +2287 kg CO₂eq).
  - A methodological–assumption matrix should be added to map system boundaries, functional units, data sources, and carbon accounting rules for each study. This would enable a more explanatory synthesis linked to observed differences.
4. **Depth of discussion**
  - Discussions repeat findings instead of engaging critically with debates. For example:
    - “Net-negative emissions” claims for biomass are presented uncritically, without considering land-use change, temporal carbon cycles, or accounting conventions.
    - Impact displacement (e.g. water use, mineral resource demand from H₂-DRI) is noted but not systematically explored.
  - The paper should highlight methodological trade-offs, standardisation needs, and offer concrete recommendations for future LCA practice and policy.

Minor Issues

- **Abstract & Plain Language Summary**: overly descriptive and partly redundant. They should clearly state number of studies, main quantitative ranges, and central conclusion (i.e. outcomes vary widely due to methodological inconsistency).
- **Figures**: Data-dense figures (3a–3f) require better integration with the text. Scenario codes are confusing; more intuitive labels are recommended.
- **Conclusion**: Needs sharper take-home messages and more specific policy implications (e.g. caution in interpreting headline GWI numbers, need for mandatory standardised reporting in LCA used for policy/funding decisions).

Overall Recommendation

**Major Revisions Required.**

 The dataset collected is useful, but the manuscript must undergo substantial restructuring and methodological strengthening. Specifically, the addition of a formal quality assessment, a methodological–assumption matrix, and a deeper, critical synthesis are essential. With these improvements, the paper could make a significant contribution to the literature on decarbonisation of steel.

Are the rationale for, and objectives of, the Systematic Review clearly stated?

Partly

Is the statistical analysis and its interpretation appropriate?

Not applicable

Are sufficient details of the methods and analysis provided to allow replication by others?

Partly

Are the conclusions drawn adequately supported by the results presented in the review?

Partly

Reviewer Expertise:

Environmental engineering; wastewater treatment; anaerobic digestion and bioenergy; life cycle assessment (LCA) of waste-to-energy and industrial processes; circular economy and sustainability assessment of resource recovery systems.

I confirm that I have read this submission and believe that I have an appropriate level of expertise to state that I do not consider it to be of an acceptable scientific standard, for reasons outlined above.

# Response

**Published:** 2025-12-23

**Authors:** Nethmi Kankanamge Dona

**Affiliations:** Institute of Environmental Sciences (CML), Leiden University, Leiden, The Netherlands

Thank you for your valuable comments. The revised version of the manuscipt that will be added shortly will contain the following changes, addressing your feedback.   

- We have expanded paragraph 8 of the introduction to discuss existing studies and highlight the contribution of our study. The specific research gap has also been rewritten to heighten the contribution of the study in paragraphs 9  and 10 of the introduction.
- Sub research questions were adjusted and rephrased to highlight the methodological drivers.
- Paragraphs 1, 2, and 3 under the procedure of the review section of the methods have been extended with several justifications for the search strategy, the database selection, and grey literature exclusion.  
- The sentence ‘All records were screened by a single reviewer (the first author) following these pre-defined exclusion and inclusion criteria. The screening process of the articles can be accessed at the extended data for further clarity’ has been added to the 7 ^th^ paragraph under the procedure of the review section to elaborate on the reviewer roles.
- A quality appraisal of the 21 studies was conducted using the Life Cycle Initiative standard and can be accessed in the extended data for the study. This is mentioned in the main text in paragraph 8 under the procedure of the review section.
- Multiple corrections have been made to the LCIA section of the results in order to strengthen the content and explain the variability.  
- A methodological matrix has been added to the extended data to support the information in the main text. A sentence saying ‘A separate methodological matrix of how data was gathered, under what criteria for this results section can be accessed at the extended data for further reference’ has been added to the beginning of the results section.
- Several clarifying sentences, such as ‘Therefore, a set of guidelines is needed on how the credits and the biogenic carbon emissions should be implemented in an LCA software, depending on the replacement ratios of biomass-based materials to fossil-based materials.’ and For example, in biomass-based studies, it can be noted that the impact on land and water use change must be assessed before ensuring a continuous supply of biomass feedstock. Land use and water use are two potential indicators that will increase in the case of cultivating biomass using existing land area for industrial purposes. It should also be noted that unless the biomass has been extracted from waste and residues, it cannot necessarily be considered biogenic, as the biomass extracted from harvested sites is either incinerated or disposed of into the atmosphere, with extractable energy present in it.  There are also other clarifiers added in the biomass section of the discussion section, clarifying these claims further.
- The sentence, such as ‘The detailed modeling in simulation software, harnessed together with field data and literature values, with correct assumptions, will benefit the completeness of the life cycle inventory immensely in biomass-based LCAs’ has been added to address this point.
- Both the abstract and the plain language summary were rewritten to address these points.
- A clarifying sentence guiding the reader to the table has been added at the end of each figure to avoid confusion.  
- The conclusion will be updated to include clearer take-home messages and discuss the implications of our findings.

# Reviewer report: Reviewer response for version 1

**DOI:** 10.21956/openreseurope.22418.r59230

**Published:** 2025-09-04

**Authors:** Manjunatha M

**Affiliations:** 1 GITAM University, Bangalore, Karnataka, India

- The abstract is overly descriptive; it should more clearly state the research gap, methodological rigor, and novelty of the systematic review beyond aggregation.
- The plain language summary largely repeats the abstract—consider shortening and focusing on policy/practical implications for broader audiences.
- The introduction is comprehensive but unfocused; it lacks a sharp articulation of the specific gap this review fills compared to existing reviews on LCA in steelmaking.
- Regional decarbonization projects (ULCOS, COURSE50, POSCO, etc.) are summarized, but the link to the systematic review’s research objectives is weak—clarify their relevance.
- The research questions are listed but not clearly mapped to the subsequent results/discussion structure. Ensure explicit alignment between questions and findings.
- The PRISMA methodology is mentioned, but transparency is limited: exact inclusion/exclusion criteria, screening procedures, and reviewer roles should be elaborated.
- The database search strategy lacks detail—expand on why only Web of Science and Scopus were selected, and discuss potential bias from excluding other databases (e.g., Google Scholar, Embase).
- The keyword string seems restrictive (“LCA,” “Hydrogen,” “Biomass”)—justify why broader terms (e.g., “footprint,” “sustainability assessment”) were excluded.
- The study screening and selection flow is presented but lacks critical information on inter-rater reliability and potential reviewer bias in exclusion.
- The results section is descriptive and case-specific but does not sufficiently synthesize findings across regions/technologies in a comparative matrix.
- Figures 2–3 contain large amounts of information but are not fully integrated into the narrative; more explicit interpretation of what the graphs reveal is needed.
- The discussion highlights heterogeneity but does not adequately explain why results diverge (e.g., methodological assumptions, system boundaries, regional contexts).
- The claim of “negative emissions” from biomass integration needs careful qualification—current presentation risks overstating results without addressing carbon accounting controversies.
- The limitations section is underdeveloped; it should expand on uncertainties in LCA datasets, functional unit definitions, and the exclusion of social/economic indicators.
- The conclusion is too general—it should provide sharper insights on research priorities (e.g., standardization of LCA methodology, data harmonization, or regional modeling needs).
- Policy implications are only briefly mentioned—expand on how the findings could inform EU decarbonization strategies, steel industry roadmaps, or carbon neutrality targets.
- The article cites numerous references, but there is a lack of critical appraisal of study quality—add an assessment of methodological robustness of the included LCAs.

Are the rationale for, and objectives of, the Systematic Review clearly stated?

Yes

Is the statistical analysis and its interpretation appropriate?

Partly

Are sufficient details of the methods and analysis provided to allow replication by others?

Yes

Are the conclusions drawn adequately supported by the results presented in the review?

Partly

Reviewer Expertise:

Sustainable Materials, Sustainablity, Custainable Materials, Building Materials and Life cycle assessment

I confirm that I have read this submission and believe that I have an appropriate level of expertise to confirm that it is of an acceptable scientific standard, however I have significant reservations, as outlined above.

# Response

**Published:** 2025-12-23

**Authors:** Nethmi Kankanamge Dona

**Affiliations:** Institute of Environmental Sciences (CML), Leiden University, Leiden, The Netherlands

Thank you for your valuable comments. The revised version of the manuscript that will be added shortly will contain the following corrections.

- The abstract was rewritten to address these points.
- The plain language summary was rewritten to address these points.
- We have expanded paragraph 8 of the introduction to discuss existing studies and highlight the contribution of our study. The specific research gap has also been rewritten to heighten the contribution of the study in paragraphs 9  and 10 of the introduction.  
- Paragraph number 2, which discusses the regional decarbonization projects, has been extended to connect with the research objectives of the study.  
- The research questions have been rephrased to enhance the alignment of the questions with the findings. The corrected research questions can be found under the sub-research questions listed in the methods section.
- The beginning of the results section contains a quick introduction that connects each section to the relevant research question.  
- Paragraphs 1, 2, and 3 under the procedure of the review section of the methods have been extended with several justifications for the inclusion and exclusion criteria, screening procedures. The reviewer roles have been elaborated in paragraph 7.
- A sentence has been added to paragraph one under the procedure of the review stating that Google Scholar was avoided due to its over-inclusivity and major overlaps with the WoS and Scopus. Unfortunately, Embase is a biomedical journal that has no relevance to LCA or the current scope of the study.
- The paragraph 2 under the procedure of the review, details have been added to justify the current search string. There are also justifications as to why the broader terms, such as ‘footprint’ and ‘sustainability assessment,’ were excluded. 
- The reviewer roles have been elaborated in paragraph 7.
- A methodological matrix has been added to the extended data to support the information in the main text. A sentence saying ‘A separate methodological matrix of how data was gathered, under what criteria can for this results section can be accessed at the extended data for further reference’ has been added to the beginning of the results section.
- Multiple corrections have been made to the results section of the results in order to strengthen the content and interpret the results.
- Several changes have been made to the discussion section to highlight the methodological assumptions, system boundaries, and regional contexts.
- Several clarifying sentences, such as ‘Therefore, a set of guidelines is needed on how the credits and the biogenic carbon emissions should be implemented in an LCA software, depending on the replacement ratios of biomass-based materials to fossil-based materials.’ and 'For example, in biomass-based studies, it can be noted that the impact on land and water use change must be assessed before ensuring a continuous supply of biomass feedstock. Land use and water use are two potential indicators that will increase in the case of cultivating biomass using existing land area for industrial purposes. It should also be noted that unless the biomass has been extracted from waste and residues, it cannot necessarily be considered biogenic, as the biomass extracted from harvested sites is either incinerated or disposed of into the atmosphere, with extractable energy present in it.'  There are also other clarifiers added in the biomass section of the discussion section, clarifying these claims further.
- The limitations section will be expanded to include the uncertainties in LCA, functional unit, and system boundary definitions, and socio-economic indicators.
- The conclusion will be rephrased to heighten the take-home messages from the study.
- The current study is not focused on the EU decarbonization, as the study refers to case studies globally. Therefore, no additional improvements were added to discuss EU steel decarbonization. 
- A quality appraisal of the 21 studies was conducted using the Life Cycle Initiative standard and can be accessed in the extended data for the study. This is mentioned in the main text in paragraph 8 under the procedure of the review section.
